# Numerosol A–D, New Cembranoid Diterpenes from the Soft Coral *Sinularia* *numerosa*

**DOI:** 10.3390/md12063371

**Published:** 2014-06-03

**Authors:** Yen-Ju Tseng, Yuan-Chien Yang, Shang-Kwei Wang, Chang-Yih Duh

**Affiliations:** 1 Department of Marine Biotechnology and Resources, National Sun Yat-sen University, Kaohsiung 804, Taiwan; E-Mails: pit0424@yahoo.com.tw (Y.-J.T.); m995020028@student.nsysu.edu.tw (Y.-C.Y.); 2 Department of Microbiology, Kaohsiung Medical University, Kaohsiung 807, Taiwan; E-Mail: skwang@cc.kmu.edu.tw; 3 Asia-Pacific Ocean Research Center, National Sun Yat-sen University, Kaohsiung 804, Taiwan; 4 Graduate Institute of Natural Products, Kaohsiung Medical University, Kaohsiung 807, Taiwan

**Keywords:** soft coral, *Sinularia numerosa*, numerosol A–D, gibberoketosterol, cytotoxicity

## Abstract

Four new cembrane-type diterpenes; numerosol A–D (**1**–**4**); along with a known steroid; gibberoketosterol (**5**); were isolated from the Taiwanese soft coral *Sinularia numerosa*. The structures of these metabolites were determined by extensive analysis of spectroscopic data. Gibberoketosterol (**5**) exhibited cytotoxicity against P-388 (mouse lymphocytic leukemia) cell line with an ED_50_ of 6.9 μM.

## 1. Introduction

**Figure 1:**
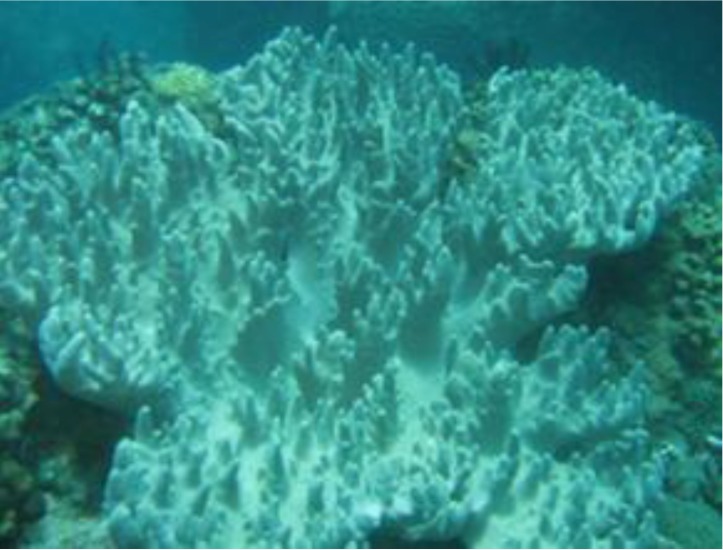
Soft coral *Sinularia numerosa*.

**Figure 2:**
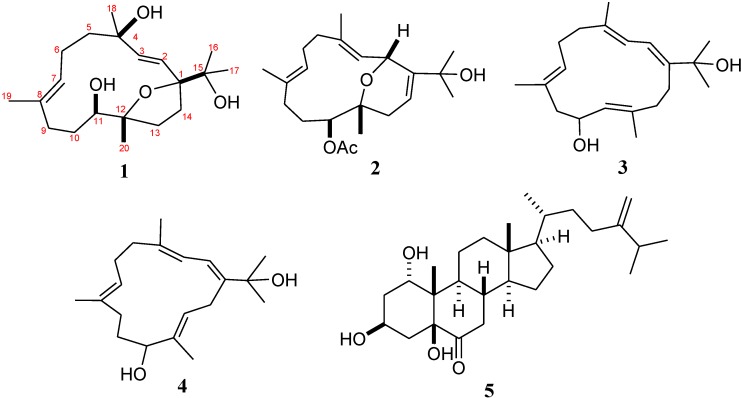
Structures of Metabolites **1**–5 *.

Soft corals belonging to the genus *Sinularia* have proven to be a rich source of diterpenes, sesquiterpenes, steroids, steroidal glycosides, sphingosine derivatives, glycolipids, and spermidine derivatives [1,2]. We have previously discovered a series of farnesyl quinols [3], sesquiterpenoids [4], norditerpenoids [5], diterpenoids [6,7], biscembranoid [8], and furanosesquiterpenoids [9] from *Sinularia* sp. Our recent investigation of natural metabolites from the soft coral *Sinularia numerosa* (Tixier-Durivault, 1970) (Figure 1), has led to the isolation of four new cembranoids, numerosols A–D (**1**–**4**) (Figure 2) and a known steroid, gibberoketosterol (**5**) [10]. The structures of numerosols A–D (**1**–**4**) were established by extensive spectroscopic analysis. The anti-human cytomegalovirus (anti-HCMV) activity of compounds **1**–**5** and its cytotoxicity against P-388 (mouse lymphocytic leukemia), HT-29 (human colon adenocarcinoma), and A-549 (human lung carcinoma) cancer cell lines were evaluated *in vitro*.

* Structures **2**–**4** are relative structures.

## 2. Results and Discussion

Numerosol A (**1**) was isolated as a colorless oil, [α]_D_^25^ −28.2 (*c* 0.4, CHCl_3_). HRESIMS, ^13^C-NMR, and DEPT spectroscopic data established the molecular formula of **1** as C_20_H_34_O_4_. The IR spectrum of **1** indicated the presence of the hydroxy functionality (ν_max_ 3413 cm^−1^). The ^1^H-NMR spectrum of **1** also showed signals for three olefinic protons at δ 5.94 (d, *J* = 15.6 Hz, H-3), 5.63 (d, *J* = 15.6 Hz, H-2), and 5.20 (d, *J* = 9.6 Hz, H-7) ppm; one oxymethine proton at δ 3.56 (d, *J* = 9.6 Hz, H-11); and an olefinic methyl group at δ 1.69 (s, H_3_-19). The spectral data of **1** indicated some similarities to those of (2*E*,7*E*)-4,11-dihydroxy-1,12-oxidocembra-2,7-diene [11], except for the data correspoding to C-15. The ^1^H–^1^H COSY spectrum exhibited correlations from H-2 to H-3, H_2_-5 to H-7, H_2_-9 to H-11, and H_2_-13 to H_2_-14. These spectroscopic findings and the requirement for four degrees of unsaturations indicated that **1** was a 14-membered cembrane-type diterpene skeleton with an ether ring.

**Figure 3:**
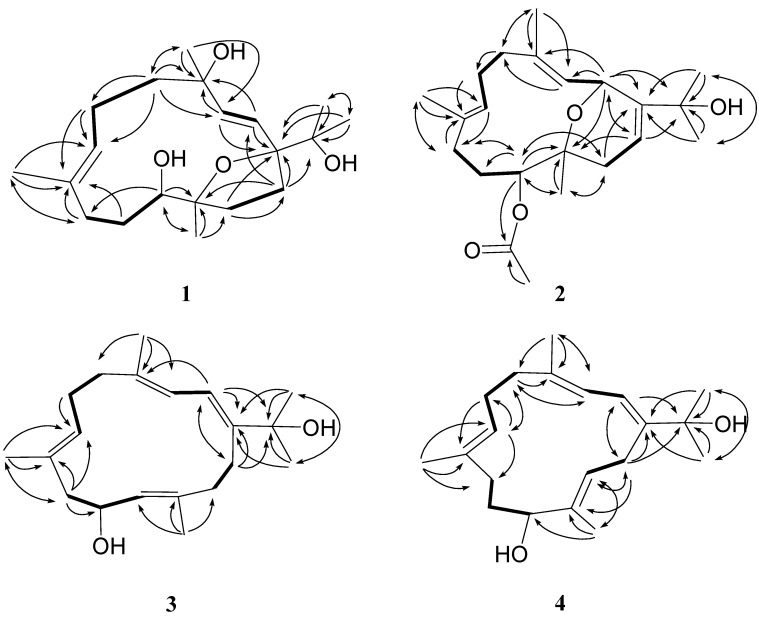
Selected ^1^H−^1^H COSY (▬) and HMBC (**→**) correlations of **1**–**4**.

**Figure 4:**
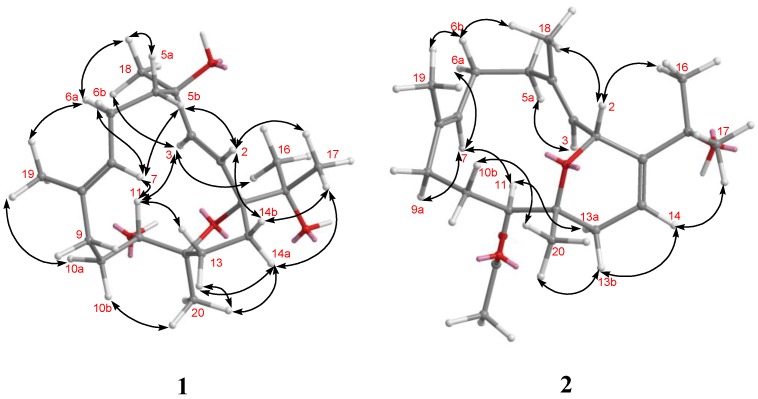
Key NOESY Correlations for **1** and **2**.

**Figure 5:**
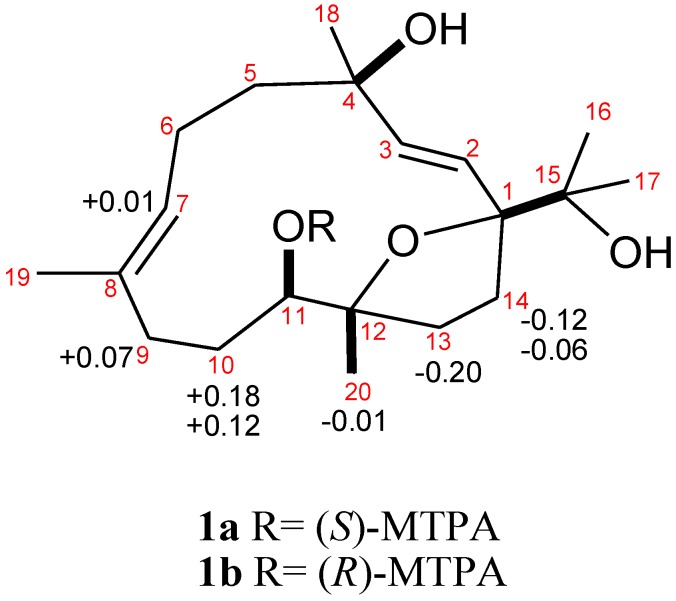
Absolute stereochemistry of **1**: Δδ*_S−R_* values in ppm for MTPA esters **1a** and **1b**.

The HMBC correlations are shown in Figure 3. NOESY correlations between H-7 and H_2_-9, and the chemical shift values at δ_C_ 16.2 (C-19) disclosed the *E* configuration for the trisubstituted olefin [10]. The *J* values for both H-2 and H-3 (15.6 Hz) further confirmed the presence of a *trans* 1,2-disubstituted double bond at C-2. NOESY correlations (Figure 4) observed between H-2 and H_3_-17, H_3_-17 and H-14a, H-14a and H_3_-20, H-3 and H_3_-16/H_3_-18, H_3_-18 and H-6a, H_3_-19 and H-6a/H-10, and H-7 and H-5b/H-6b/H-11 indicated the relative configurations for the 14-membered ring carbons, which were identical to those of (2*E*,7*E*)-4,11-dihydroxy-1,12-oxidocembra-2,7-diene. Analysis of the Δδ*_S−R_* values (Figure 5) according to the Mosher model indicated an *R* configuration for C-11 of **1** based on the deshielded nature of H-10, H-9, and H-7 of the (*S*)-MTPA ester **1a**. Therefore, the absolute stereochemistry of Numerosol A (**1**) was established as (1*R*,4*R*,11*R*,12*S*,2*E*,7*E*)-4,11,15-trihydroxy-1,12-oxidocembra-2,7-diene.

HRESIMS of Numerosol B (**2**) exhibited a pseudo-molecular ion peak at *m/z* 385.2353 [M + Na]^+^, consistent with the molecular formula of C_22_H_34_O_4_, requiring six degrees of unsaturation. The IR spectrum of **2** revealed the presence of hydroxy (ν_max_ 3440 cm^−1^) and carbonyl (ν_max_ 1730 cm^−1^) moieties. The ^13^C-NMR spectrum of **2** (Table 1) displayed 22 carbon signals, and a DEPT experiment confirmed the presence of six methyls, five methylenes, five methines, and six quaternary carbons. The presence of six carbon signals at δ 150.3 (qC), 138.8 (qC), 134.7 (qC), 126.2 (CH), 125.7 (qC), and 115.3 (CH) and the three proton signals at δ 5.83 (1H, dd, *J* = 7.5, 2.5 Hz), δ 5.35 (1H, d, *J* = 10.5 Hz), and δ 5.24 (1H, dd, *J* = 11.0, 3.0 Hz), were attributable to three trisubstituted double bonds. Moreover, an acetoxy group was determined based on NMR signals at δ_C_ 171.7 (qC), 21.3 (CH_3_), and δ_H_ 2.10 (3H, s). The ^1^H-NMR spectrum also displayed two hydroxy-containing methine signals (δ 5.39, d, *J* = 9.0 Hz; δ 5.01, d, *J* = 10.5 Hz). Thus, the bicyclic structure of **2** was revealed. From the ^1^H–^1^H COSY spectrum (Figure 3), it was possible to identify four different structural units (Figure 3). Key HMBC correlations of H-2 to C-1, C-3, C-4, C-12, and C-15; H-3 to C-5; H_2_-5 to C-6 and C-18; H-6 to C-8; H-7 to C-9; H-11 to C-9, C-10, C-12, C-13, C-20, and 11-OAc; H_2_-13 to C-1, C-11 and C-20; H-14 to C-2 and C-15; H_3_-16 to C-1, C-15, and C-17; H_3_-17 to C-1, C-15, and C-16; H_3_-18 to C-3, C-4, and C-5; H_3_-19 to C-7, C-8, and C-9; H_3_-20 to C-11, C-12, and C-13; H_3_-OAc to 11-OAc permitted the establishment of the cembrane-type skeleton of **2**. The *E* geometry of the double bonds at C-3/C-4, C-7/C-8, and C-14/C-1 was supported by NOE correlations between H-3 and H-5a (δ 2.08, m), between H-7 and H-9a (δ 1.82, m), and between H-14 and H_3_-17 (Figure 4). The chemical shift values at δ_C_ 15.2 (C-18) and δ_C_ 16.4 (C-19) further supported the *E* configuration at C-3/C-4, C-7/C-8 [10]. The β-orientation of H-2 was established from NOE correlations observed between H-2 and H_3_-18, H_3_-18 and H-6b (δ 2.43, dd, *J* = 11.0, 3.0 Hz), and H-6b and H_3_-19. The NOE correlations observed between H-6a (δ 2.08, m) and H-7, H-7 and H-11, H-11 and H-13a (δ 2.22, m), reflected the α-orientation of H-11. Also, the NOE correlations observed between H-13b (δ 2.14, m) and H_3_-20, reflected the β-orientation of H_3_-20. According to the above NOE correlations, and the others shown in Figure 4, the relative configurations at C-2, C-11, and C-12 for **2** were determined as 2R*,11S*, and 12R*, respectively.

**Table 1:** ^1^H and ^13^C-NMR Spectroscopic Data for compounds **1** and **2**.

Position	1		2		
	δ_H_^a^	δ_C_^b^	δ_H_^c^		δ_C_^d^
1		91.3, qC *^f^*		150.3, qC	
2	5.63 d (15.6) *^e^*	128.2, CH	5.01 d (10.5)	69.2, CH	
3	5.94 d (15.6)	138.4, CH	5.35 d (10.5)	126.2, CH	
4		74.5, qC		138.8, qC	
5	a:1.85 m; b:1.59 m	43.9, CH_2_	a:2.08 m; b:2.20 m	39.7, CH_2_	
6	a:2.27 m; b:2.17 m	24.35, CH_2_	a:2.08 m; b:2.43 dd (11.0, 3.0);	26.1, CH_2_	
7	5.20 d (9.6)	129.2, CH	5.24 dd (11.0, 3.0)	125.7, CH	
8		133.5, qC		134.7, qC	
9	2.10 m	35.4, CH_2_	a:1.82 t (3.0); b:1.94 m	35.4, CH_2_	
10	a:1.91 dd (14.7, 9.6); b:1.36 d (14.7)	29.4, CH_2_	a:1.82 m; b:1.44 m	26.5, CH_2_	
11	3.56 d (9.6)	76.4, CH	5.39 d (9.0)	78.8, CH	
12		85.4, qC		73.6, qC	
13	1.79 m	36.6, CH_2_	a:2.22 m; b:2.14 m	32.4, CH_2_	
14	a:2.43 m; b:1.69 m	31.0, CH_2_	5.83 dd (7.5, 2.5)	115.3, CH	
15		72.4, qC		71.8, qC	
16	1.07 s	25.9, CH_3_	1.31 s	28.8, CH_3_	
17	1.14 s	24.41, CH_3_	1.31 s	28.9, CH_3_	
18	1.28 s	28.4, CH_3_	1.74 s	15.2, CH_3_	
19	1.69 s	16.2, CH_3_	1.59 s	16.4, CH_3_	
20	1.12 s	19.4, CH_3_	1.05 s	23.3, CH_3_	
OAc-11			2.10 s	21.3, CH_3_	
				171.7, qC	

^a^ Spectra recorded at 400 MHz in CDCl_3_; ^b^ Spectra recorded at 100 MHz in CDCl_3_; ^c^ Spectra recorded at 500 MHz in CDCl_3_; ^d^ Spectra recorded at 125 MHz in CDCl_3_; ^e^ *J* values (in Hz) are in parentheses; ^f^ Carbon types are deduced by HSQC and DEPT experiments.

**Figure 6:**
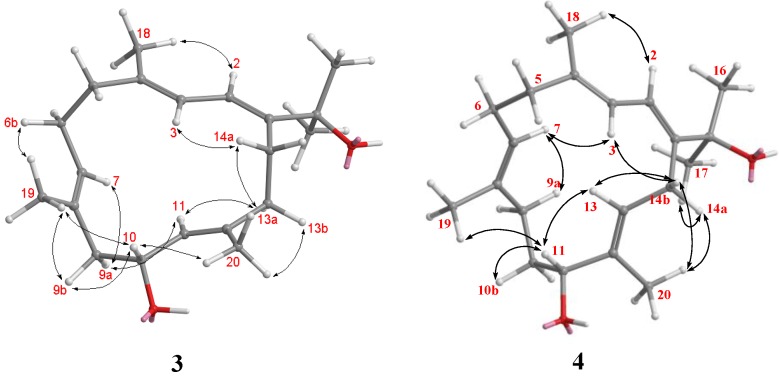
Key NOESY Correlations for **3** and **4**.

Numerosol C (**3**) was obtained as colorless oil. Its HRESIMS (*m/z* 327.2299 [M + Na]^+^) established the molecular formula C_20_H_32_O_2_, requiring five degrees of unsaturation. The IR spectrum of **3** revealed the presence of a hydroxyl (ν_max_ 3404 cm^−1^) moiety. Analysis of the ^1^H–^1^H COSY and HMBC correlations (Figure 3) were diagnostic in determining that the planar framework of numerosol C, having a 14-membered ring, was proposed as **3**. The observed COSY correlation between H-10 and H-11 and the key HMBC correlations from H-11 to C-10 confirmed the location of the hydroxyl group. The NMR data (Table 2) of **3** were similar to those of gibberosene G [12]. However, resonances for the methane proton at C-15 in gibberosene G were absent from the ^1^H-NMR spectrum of **3**. In addition, the methine carbon at δ 34.4 in gibberosene G was downfield-shifted to δ 74.1 in **3**. Thus, the methine proton in gibberosene G should be replaced by a hydroxyl group in **3**. The *E*-geometries of the four double bonds at C-1/C-2, C-3/C-4, C-7/C-8, and C-12/C-13 were determined by the NOE interactions (Figure 6) displayed by the methyl protons at H_3_-16 with H-2, H-2 with H_3_-18, H-7 with H_2_-9a, and H-11 with H-13a, respectively. The chemical shift values at δ_C_ 18.0 (C-18), δ_C_ 16.7 (C-19), and δ_C_ 15.9 (C-20) also supported the *E* configuration at C-3/C-4, C-7/C-8, and C-11/C-12 [10]. The relative structure of numerosol C (**3**) was established as (−)-(1*E*,3*E*,7*E*,12*E*)-10-hydroxycembra-1,3,7,12-tetraene. Due to the decomposition of compound **3**, the stereo center present in compounds **3** could not be conclusively assigned.

**Table 2:** ^1^H and ^13^C-NMR Spectroscopic Data for compounds **3** and **4**.

Position	3		4	
	δ_H_^a^	δ_C_^b^	δ_H_^a^	δ_C_^b^
1		147.1, qC *^d^*		145.5, qC
2	6.39 d (11.6) *^c^*	118.7, CH	6.37 d (10.4)	118.7, CH
3	5.78 d (11.6)	120.0, CH	5.71 d (10.4)	121.3, CH
4		138.3, qC		137.4, qC
5	2.18 m	38.4, CH_2_	2.15 m	38.0, CH_2_
6	a:2.24 m; b:2.18 m;	24.5, CH_2_	2.16 m	24.5, CH_2_
7	5.02 d (5.2)	127.8, CH	4.95 brs	126.0, CH
8		131.2, qC		133.4, qC
9	a:2.10 dd (12.4, 10.8); b:2.46 m	48.0, CH_2_	a:2.13 m; b:2.00 m	35.9, CH_2_
10	4.55 ddd (14.8, 9.6, 4.0)	66.2, CH	a:1.85 m; b:1.74 m	29.4, CH_2_
11	5.13 d (9.6)	128.4, CH	3.93 dd (10.4, 3.6)	79.1, CH
12		140.2, qC		135.5, qC
13	a:1.92 m; b:2.42 m	42.4, CH_2_	5.20 t (5.2)	130.4, CH
14	a:2.22 m b:2.40 m	26.0, CH_2_	a:2.83 dd (16.8, 5.2) b:3.00 dd (16.8, 5.2)	26.7, CH_2_
15		74.1, qC		73.9, qC
16	1.38 s	29.9, CH_3_	1.37 s	28.8, CH_3_
17	1.38 s	29.8, CH_3_	1.37 s	28.9, CH_3_
18	1.79 s	18.0, CH_3_	1.76 s	17.6, CH_3_
19	1.60 s	16.7, CH_3_	1.56 s	15.2, CH_3_
20	1.74 s	15.9, CH_3_	1.65 s	11.0, CH_3_

^a^ Spectra recorded at 400 MHz in CDCl_3_; ^b^ Spectra recorded at 100 MHz in CDCl_3_; ^c^ *J* values (in Hz) are in parentheses; ^d^ Multiplicities are deduced by HSQC and DEPT experiments.

Numerosol D (**4**) was found to have the molecular formula C_20_H_32_O_2_, as indicated by the HRESIMS (*m/z* found 327.2299, [M + Na]^+^), requiring five degrees of unsaturation. The ^13^C-NMR signals (Table 2) at δ_C_ 145.5 (qC, C-1), 118.7 (CH, C-2), 121.3 (CH, C-3), 137.4 (qC, C-4), 126.0 (CH, C-7), 133.4 (qC, C-8), 135.5 (qC, C-12) and 130.4 (CH, C-13) revealed four trisubstituted double bonds in **4**. The oxygen atoms were attributable to two hydroxy groups (IR 3420 cm^−1^). The first hydroxymethine proton (δ 3.93, dd, *J* = 10.4, 3.6 Hz) was located at C-11 based on COSY correlations with a set of methylene protons (δ 1.74, m and 1.85, m, H_2_-10) and a HMBC correlation from H-11 to H_3_-20 (δ 1.65, s). HMBC correlations (Figure 3) from H_3_-16 and H_3_-17 to C-15 (δ 73.9, qC) confirmed the second hydroxymethine to be located at C-15. The *E*-geometries of the four double bonds at C-1/C-2, C-3/C-4, C-7/C-8, and C-12/C-13 were determined by the following NOE interactions (Figure 6): H-15 with H-2, H-2 with H_3_-18, H-7 with H_2_-9, and H-11 with H-13, respectively. The chemical shift values at δ_C_ 17.6 (C-18), δ_C_ 15.2 (C-19), and δ_C_ 11.0 (C-20) also supported the *E* configuration at C-3/C-4, C-7/C-8, and C-2/C-3 [10]. The relative structure of numerosol D (**4**) was established as (+)-(1*E*,3*E*,7*E*,12*E*)-11-hydroxycembra-1,3,7,12-tetraene. Due to the decomposition of compound **4**, the stereo center present in compounds **4** could not be determined.

Metabolites **1**–**5** in our study were evaluated for cytotoxicity against P-388, A549, and HT-29 cancer cell lines as well as antiviral activity against human cytomegalovirus. Preliminary cytotoxic screening revealed that **5** exhibited cytotoxicity against P-388 (mouse lymphocytic leukemia) cell line with an ED_50_ of 6.9 μM.

## 3. Experimental Section

### 3.1. General Experimental Procedures

The NMR spectra were recorded on a Varian Unity INOVA 500 FT-NMR spectrometer at 500 MHz for ^1^H and 125 MHz for ^13^C or on a Varian MR 400 FT-NMR at 400 MHz for ^1^H and 100 MHz for ^13^C, respectively. ^1^H-NMR chemical shifts are expressed in δ referring to the solvent peak δ_H_ 7.27 for CDCl_3_, and coupling constants are expressed in Hz. ^13^C-NMR chemical shifts are expressed in δ referring to the solvent peak δ_C_ 77.0 for CDCl_3_. Optical rotations were determined with a JASCO P1020 digital polarimeter. IR spectra were recorded on a JASCO FT/IR4100 infrared spectrophotometer. LRMS and HRMS were obtained by ESI on a Bruker APEX ΙΙ mass spectrometer. Silica gel 60 (Merck, Darmstadt, Germany, 230–400 mesh) and LiChroprep RP-18 (Merck, 40–63 μm) were used for column chromatography. Precoated silica gel plates (Merck, Kieselgel 60 F_254_, 0.25 mm) and precoated RP-18 F_254s_ plates (Merck) were used for thin-layer chromatography analysis. High-performance liquid chromatography was carried out using a Hitachi L-7100 pump equipped with a Hitachi L-7400 UV detector at 220 nm together with a semi-preparative reversed-phase column (Merck, Hibar LiChrospher RP-18e, 5 μm, 250 × 25 mm).

### 3.2. Animal Material

The octocoral *S. numerosa* was collected by hand using scuba at Sansiantai, Taitong County, Taiwan, in August 2008, at a depth of 6–8 m and stored in a freezer until extraction. This soft coral was identified by Prof. Chang-Fong Dai, Institute of Oceanography, National Taiwan University. A voucher specimen (SST-12) was deposite in the Department of Marine Biotechnology and Resources, National Sun Yat-sen University.

### 3.3. Extraction and Separation

A specimen of *S. numerosa* (4.0 kg, wet weight) was chopped into small pieces and extracted with acetone (3L × 3) at room temperature. The combined acetone extract was then partitioned with EtOAc and H_2_O. The EtOAc-soluble residue (27.5 g) was subjected to Si 60 CC using *n*-hexane–EtOAc–MeOH mixtures of increasing polarity for elution. Fraction 19, eluted with *n*-hexane–EtOAc (1:2), was purified by reverse-phase HPLC (MeOH–H_2_O, 70:30) to afford **2** (1.2 mg, 0.00003 w/w%). Fraction 23, eluted with *n*-hexane–EtOAc (1:6), was purified by reverse-phase HPLC (MeOH–H_2_O, 70:30) to afford **3** (1.3 mg, 0.000032 w/w%) and **4** (2.4 mg, 0.00006 w/w%). Fraction 24, eluted with *n*-hexane–EtOAc (1:8), was purified by reverse-phase HPLC (MeOH–H_2_O, 75:25) to afford **1** (1.9 mg, 0.000047 w/w%).

Numerosol A (**1**): Colorless oil; [α]_D_^25^ = −28 (*c* 0.4, CHCl_3_); IR (neat) ν_max_ 3413, 2925, 2857, 1456, 1378, 1166, 1074, 952, 756, 665 cm^−1^; ^1^H and ^13^C-NMR data, see Table 1; ESIMS *m*/*z* 361 [M + Na]^+^; HRESIMS *m*/*z* 361.2357 (calcd for C_20_H_34_O_4_Na, 361.2355) (Supplementary Figures S1–S9).

Numerosol B (**2**): Colorless oil; [α]_D_^25^ = −60 (*c* 0.2, CHCl_3_); IR (neat) ν_max_ 3440, 2919, 2851, 1730, 1575, 1371, 1240, 1017 cm^−1^; ^1^H and ^13^C-NMR data, see Table 1; ESIMS *m*/*z* 385 [M + Na]^+^; HRESIMS *m*/*z* 385.2353 (calcd for C_22_H_34_O_4_Na, 385.2355) (Supplementary Figures S10–S15).

Numerosol C (**3**): Colorless oil; [α]_D_^25^ = −100 (*c* 0.3, CHCl_3_); IR (neat) ν_max_ 3404, 2922, 2853, 1564, 1417, 1255, 1016, 771 cm^−1^; ^1^H and ^13^C-NMR data, see Table 2; ESIMS *m*/*z* 327 [M + Na]^+^; HRESIMS *m*/*z* 327.2299 (calcd for C_20_H_32_O_2_Na, 327.2300) (Supplementary Figures S16–S21).

Numerosol D (**4**): Colorless oil; [α]_D_^25^ = +8 (*c* 0.4, CHCl_3_); IR (neat) ν_max_ 3420, 2972, 2931, 1564, 1419, 1381, 998, 765 cm^−1^; ^1^H and ^13^C-NMR data, see Table 2; ESIMS *m*/*z* 327 [M + Na]^+^; HRESIMS *m*/*z* 327.2299 (calcd for C_20_H_32_O_2_Na, 327.2300) (Supplementary Figures S22–S27).

### 3.4. Cytotoxicity Assay

Cytotoxicity was determined on P-388 (mouse lymphocytic leukemia), HT-29 (human colon adenocarcinoma), and A-549 (human lung epithelial carcinoma) tumor cells using a modification of the MTT colorimetric method according to a previously described procedure [13,14]. The provision of the P-388 cell line was supported by J.M. Pezzuto, formerly of the Department of Medicinal Chemistry and Pharmacognosy, University of Illinois at Chicago. HT-29 and A-549 cell lines were purchased from the American Type Culture Collection. To measure the cytotoxic activities of the tested compounds, five concentrations with three replications were performed on each cell line. Mithramycin was used as a positive control.

### 3.5. Anti-HCMV Assay

To determine the effects of natural products upon HCMV cytopathic effect (CPE), confluent human embryonic lung (HEL) cells grown in 24-well plates were incubated for 1 h in the presence or absence of various concentrations of tested natural products with three replications. Ganciclovir was used as a positive control. Then, cells were infected with HCMV at an input of 1000 pfu (plaque forming units) per well of a 24-well dish. Antiviral activity was expressed as IC_50_ (50% inhibitory concentration), or compound concentration required to reduce virus induced CPE by 50% after 7 days as compared with the untreated control. To monitor the cell growth upon treatment with natural products, an MTT-colorimetric assay was employed [15].

## 4. Conclusions

In previous studies, gibberoketosterol (**5**) showed moderate cytotoxicity and selectivity against the growth of Hepa59T/VGH cancer cells was evaluated [12]. This investigation of soft coral *S. munerosa* collected at San-Hsian-Tai (Taitong County, Taiwan) has led to the isolation of four new cembrane-type diterpenoids, Numerosol A–D (**1**–**4**) and the known steroid gibberoketosterol (**5**). Compound **5** displayed moderate cytotoxicity and selectivity against P-388, with ED_50_ of 6.9 μM.

## Supplementary Files

Supplementary File 1 Supplementary Information (PDF, 2512 KB)
