# Supplementary material for: Numerosol A–D, New Cembranoid Diterpenes from the Soft Coral Sinularia numerosa
Source: Mar Drugs. 2014 Jun 3;12(6):3371–80. doi: 10.3390/md12063371 (PMC4071581; doi:10.3390/md12063371)
Supplement: Supplementary File 1 — Supplementary Information (PDF, 2512 KB) [file marinedrugs-12-03371-s001.pdf]

Supplementary Information

Figure S1. ^{1}H NMR spectrum (400 MHz) of numerosol A (**1**) in $CDCl_3$.
Figure S2. ^{13}C NMR spectrum (400 MHz) of numerosol A (**1**) in $CDCl_3$.
Figure S3. COSY spectrum (400 MHz) of numerosol A (**1**) in $CDCl_3$.
Figure S4. COSY spectrum (upper field region) of numerosol A (**1**) in $CDCl_3$.
Figure S5. HSQC spectrum (400 MHz) of numerosol A (**1**) in $CDCl_3$.
Figure S6. HMBC spectrum (400 MHz) of numerosol A (**1**) in $CDCl_3$.
Figure S7. NOESY spectrum (400 MHz) of numerosol A (**1**) in $CDCl_3$.
Figure S8. COSY spectrum (400 MHz) of (*S*)-MTPA ester (**1a**) in pyridine-d_5.
Figure S9. COSY spectrum (400 MHz) of (*R*)-MTPA ester (**1b**) in pyridine-d_5.
Figure S10. ^{1}H NMR spectrum (500 MHz) of numerosol B (**2**) in $CDCl_3$.
Figure S11. ^{13}C NMR spectrum (500 MHz) of numerosol B (**2**) in $CDCl_3$.
Figure S12. COSY spectrum (500 MHz) of numerosol B (**2**) in $CDCl_3$.
Figure S13. HSQC spectrum (500 MHz) of numerosol B (**2**) in $CDCl_3$.
Figure S14. HMBC spectrum (500 MHz) of numerosol B (**2**) in $CDCl_3$.
Figure S15. NOESY spectrum (500 MHz) of numerosol B (**2**) in $CDCl_3$.
Figure S16. ^{1}H NMR spectrum (400 MHz) of numerosol C (**3**) in $CDCl_3$.
Figure S17. ^{13}C NMR spectrum (400 MHz) of numerosol C (**3**) in $CDCl_3$.
Figure S18. COSY spectrum (400 MHz) of numerosol C (**3**) in $CDCl_3$.
Figure S19. HSQC spectrum (400 MHz) of numerosol C (**3**) in $CDCl_3$.
Figure S20. HMBC spectrum (400 MHz) of numerosol C (**3**) in $CDCl_3$.
Figure S21. NOESY spectrum (400 MHz) of numerosol C (**3**) in $CDCl_3$.
Figure S22. ^{1}H NMR spectrum (400 MHz) of numerosol D (**4**) in $CDCl_3$.
Figure S23. ^{13}C NMR spectrum (400 MHz) of numerosol D (**4**) in $CDCl_3$.
Figure S24. COSY spectrum (400 MHz) of numerosol D (**4**) in $CDCl_3$.
Figure S25. HSQC spectrum (400 MHz) of numerosol D (**4**) in $CDCl_3$.
Figure S26. HMBC spectrum (400 MHz) of numerosol D (**4**) in $CDCl_3$.
Figure S27. NOESY spectrum (400 MHz) of numerosol D (**4**) in $CDCl_3$.

Figure S1. ^{1}H NMR spectrum (400 MHz) of numerosol A (**1**) in $CDCl_3$.

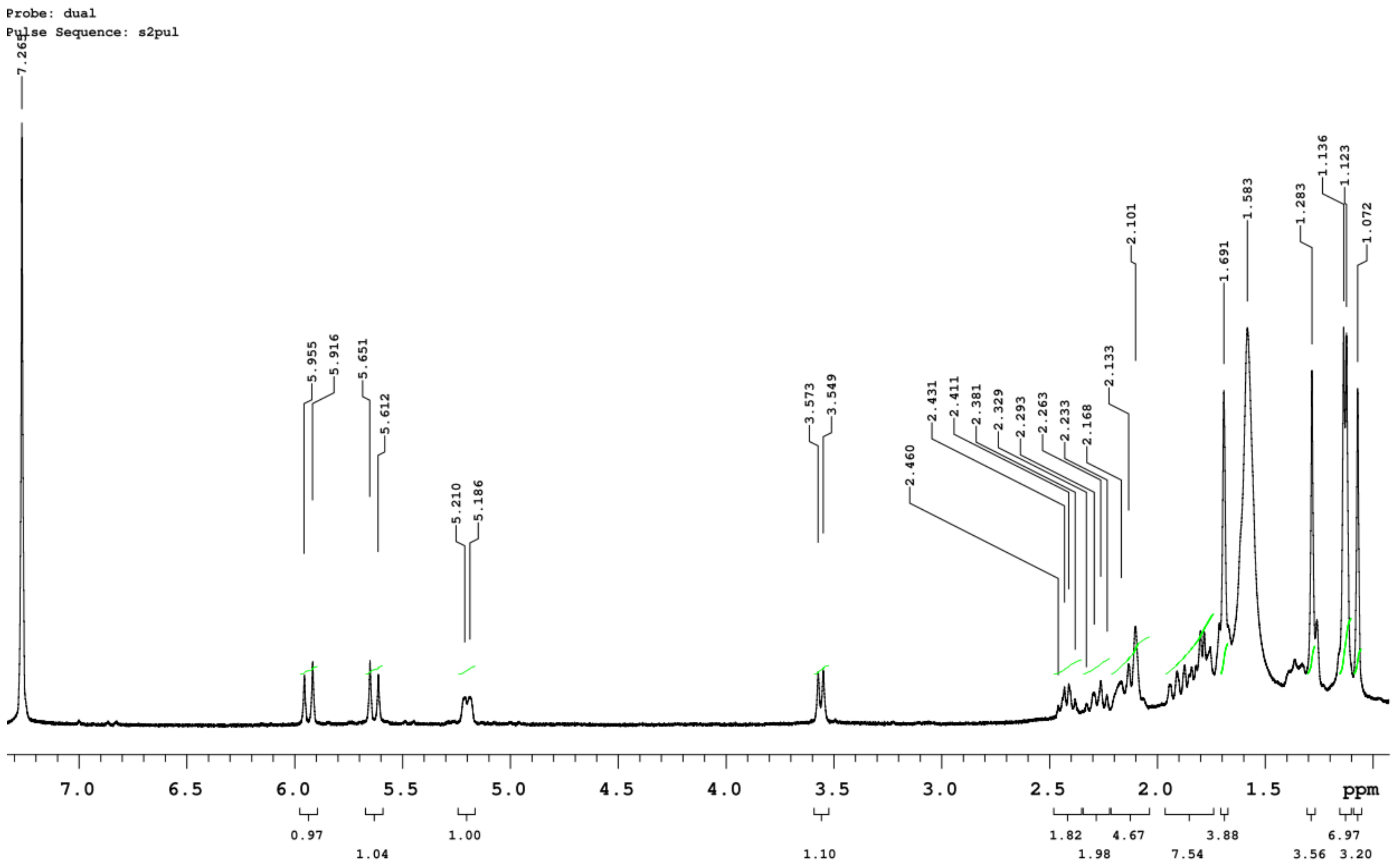

Figure S2. ^{13}C NMR spectrum (400 MHz) of numerosol A (**1**) in $CDCl_3$.

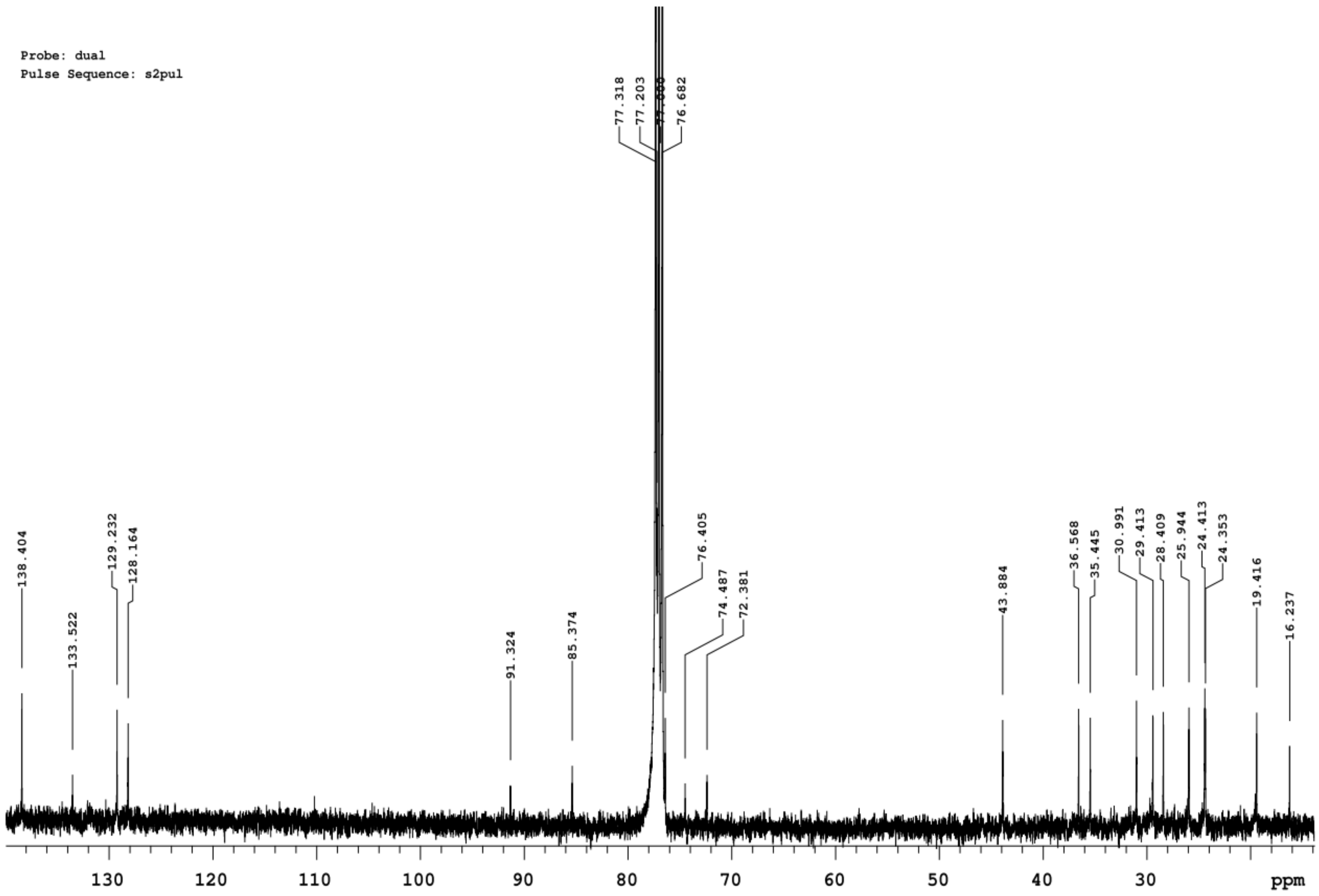

Figure S3. COSY spectrum (400 MHz) of numerosol A (**1**) in $CDCl_3$.

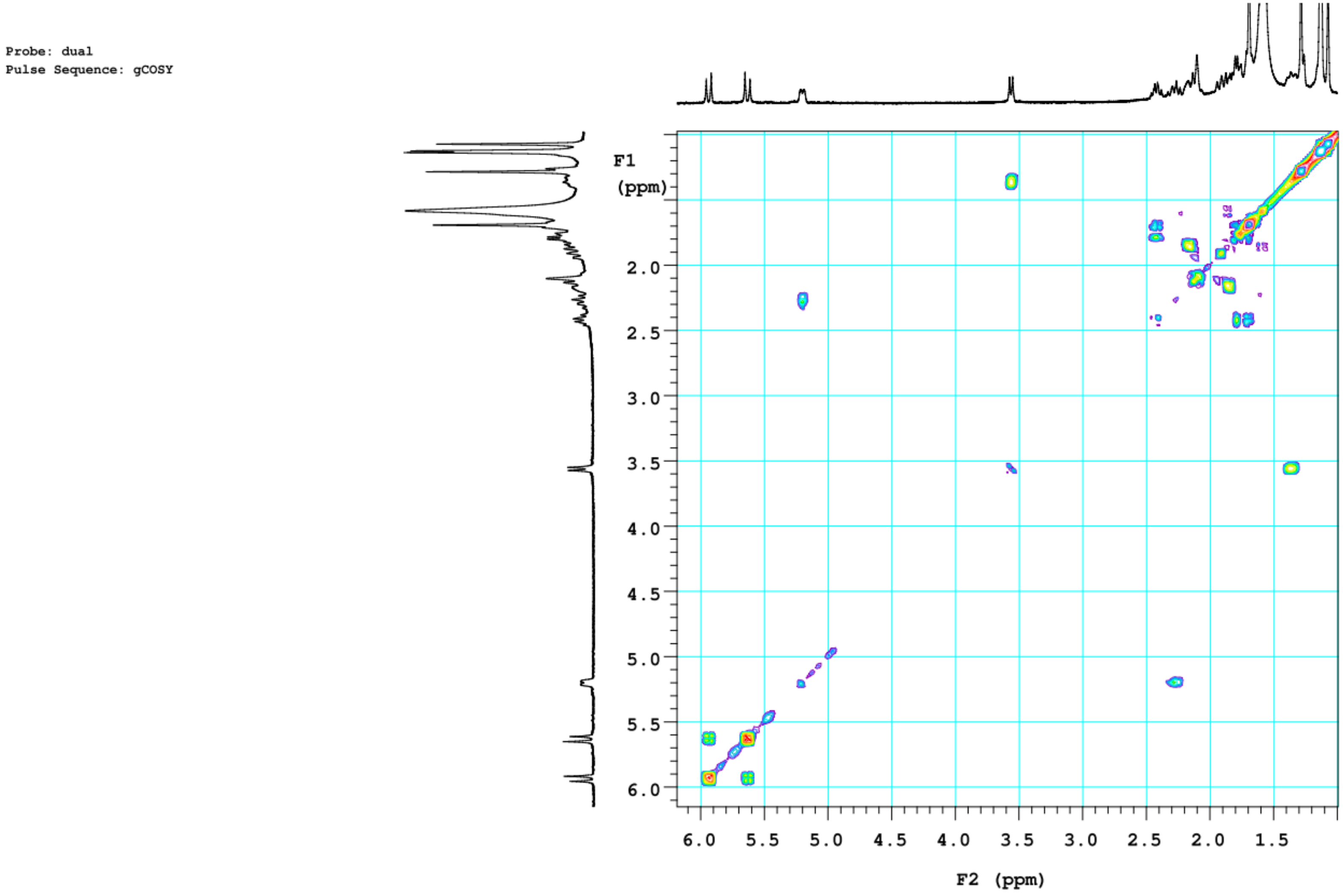

Figure S4. COSY spectrum (upper field region) of numerosol A (**1**) in $CDCl_3$.

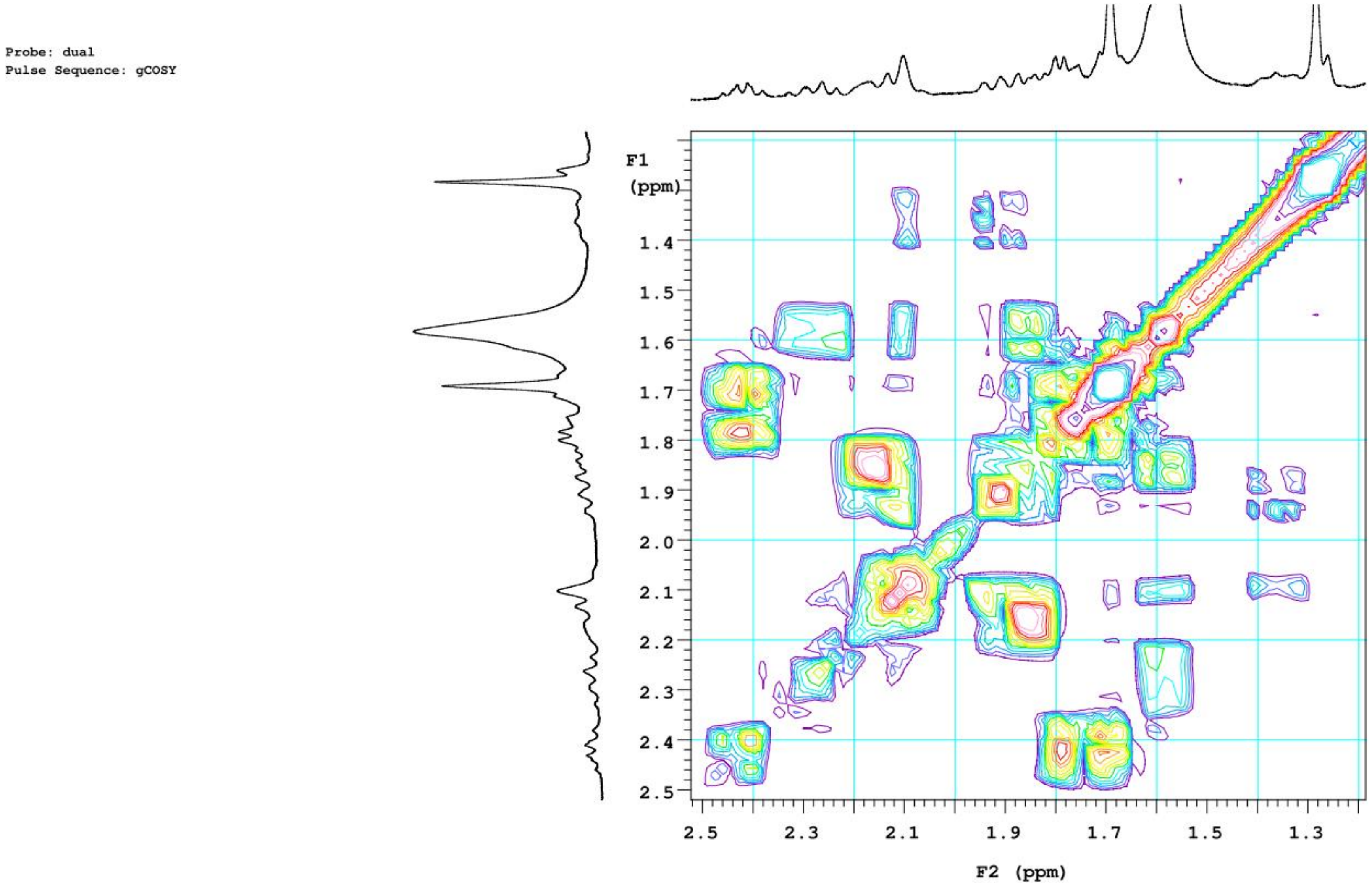

Figure S5. HSQC spectrum (400 MHz) of numerosol A (**1**) in $CDCl_3$.

Probe: dual
Pulse Sequence: gHSQCAD

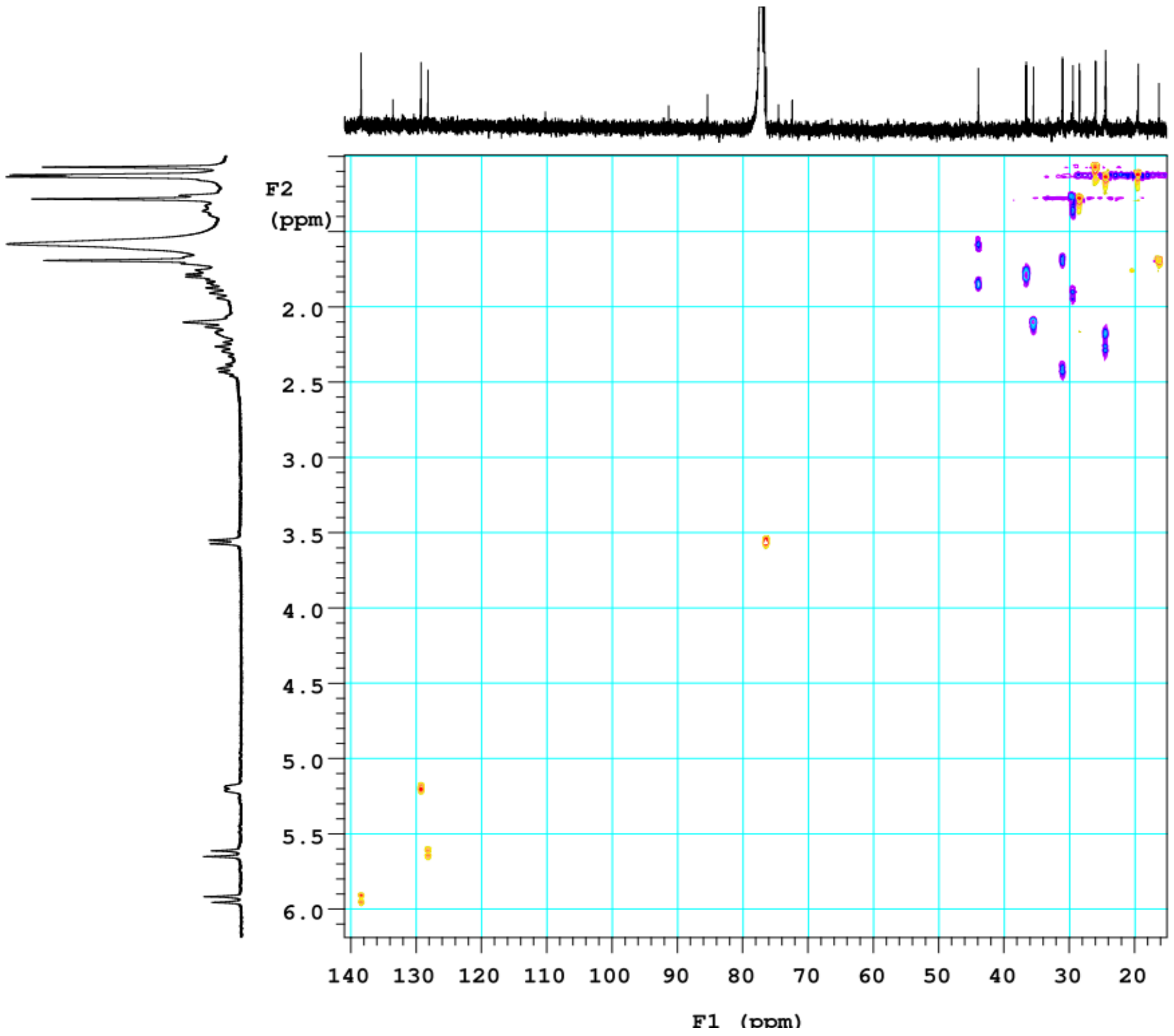

Figure S6. HMBC spectrum (400 MHz) of numerosol A (**1**) in $CDCl_3$.

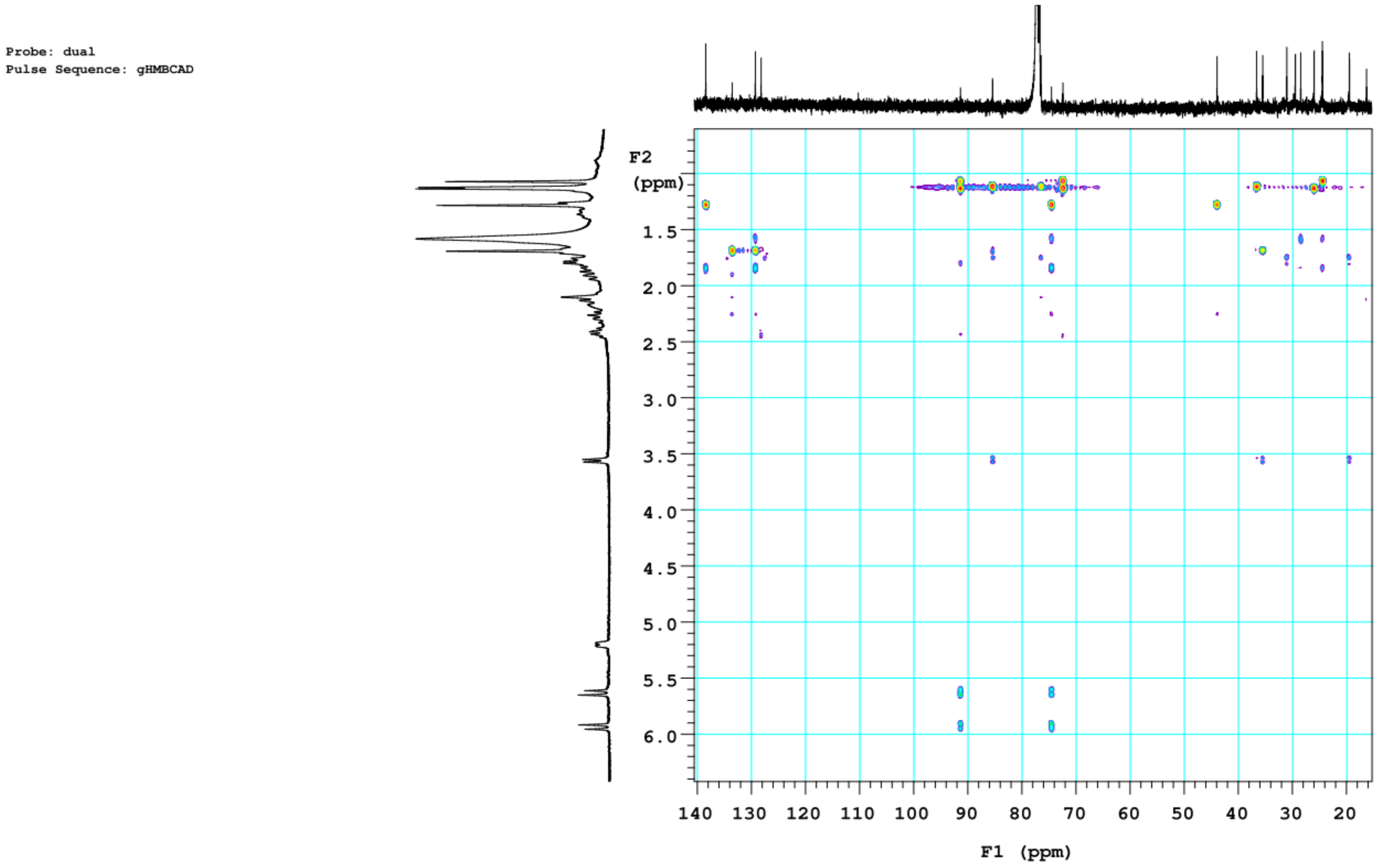

Figure S7. NOESY spectrum (400 MHz) of numerosol A (**1**) in $CDCl_3$.

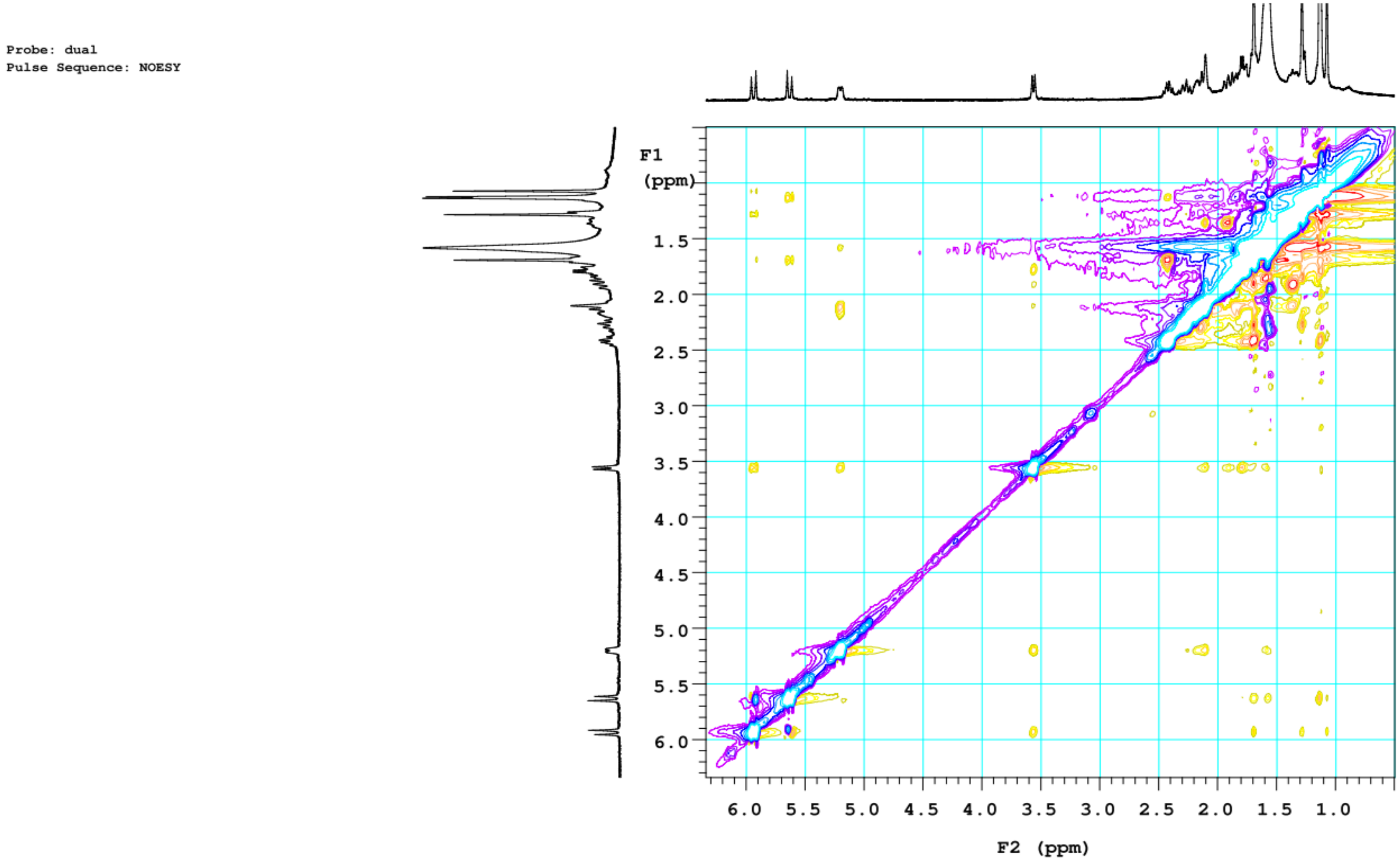

Figure S8. COSY spectrum (400 MHz) of (*S*)-MTPA ester (**1a**) in pyridine-d_5.

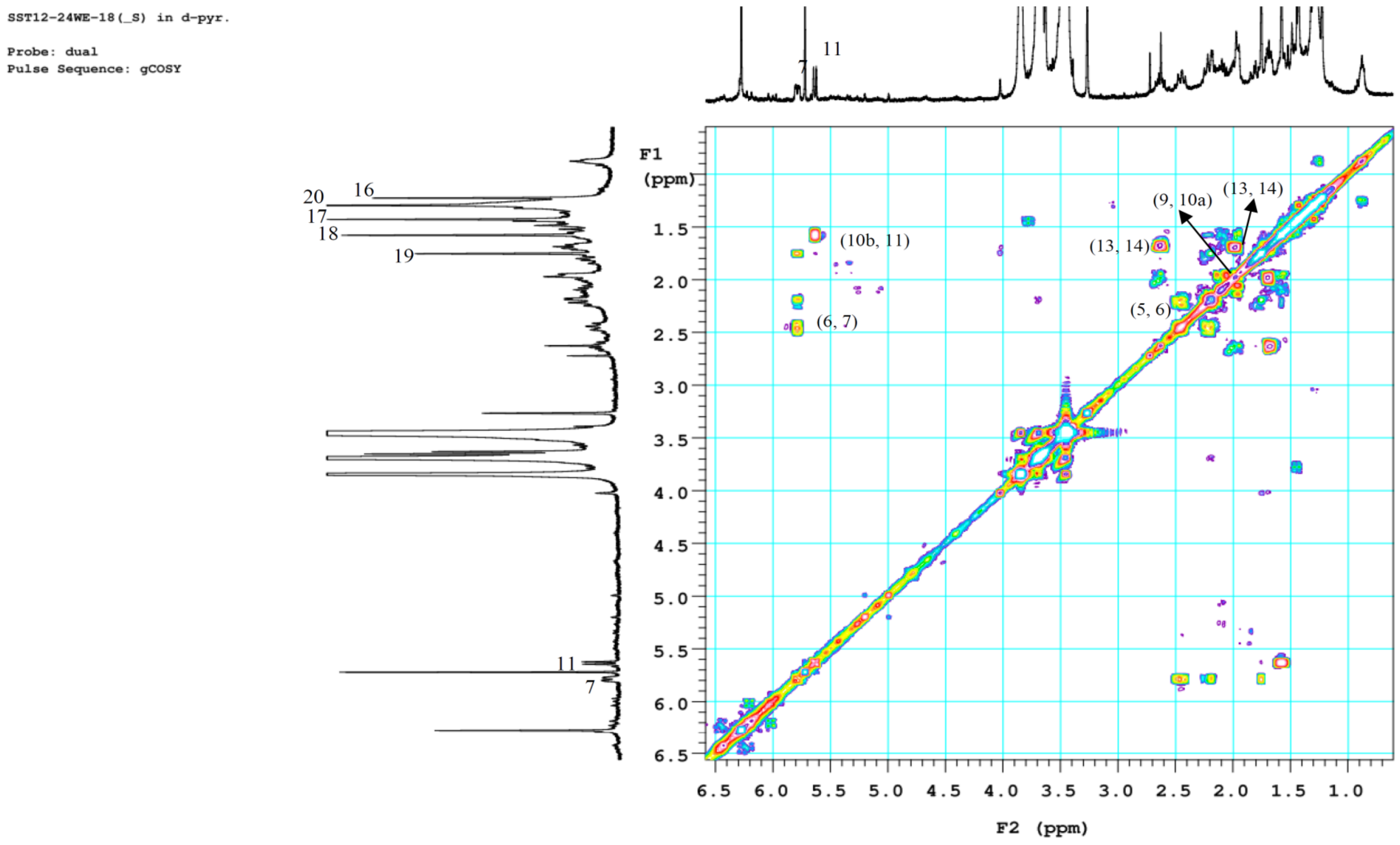

Figure S9. COSY spectrum (400 MHz) of (*R*)-MTPA ester (**1b**) in pyridine-d_5.

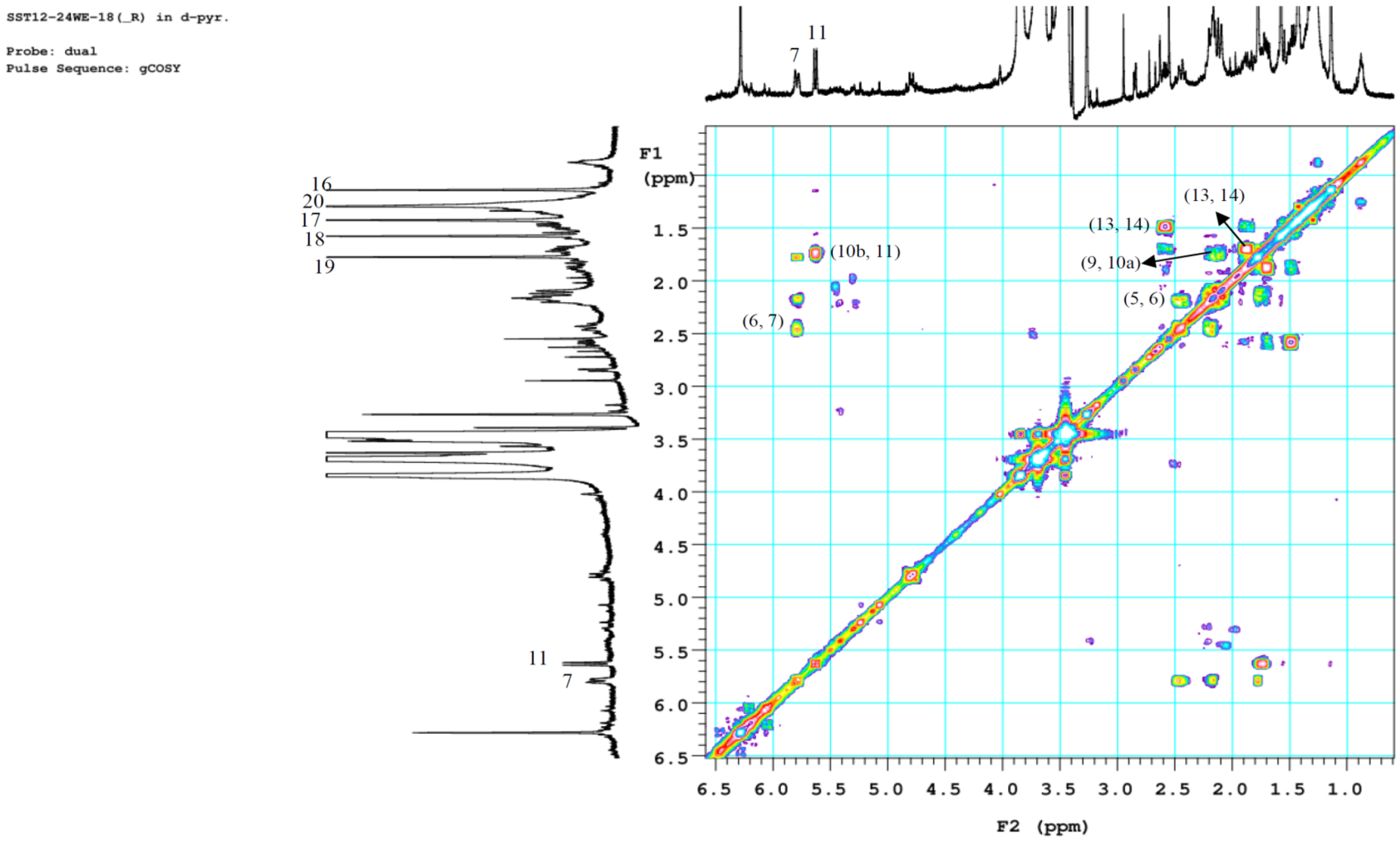

Figure S10. ^{1}H NMR spectrum (500 MHz) of numerosol B (**2**) in $CDCl_3$.

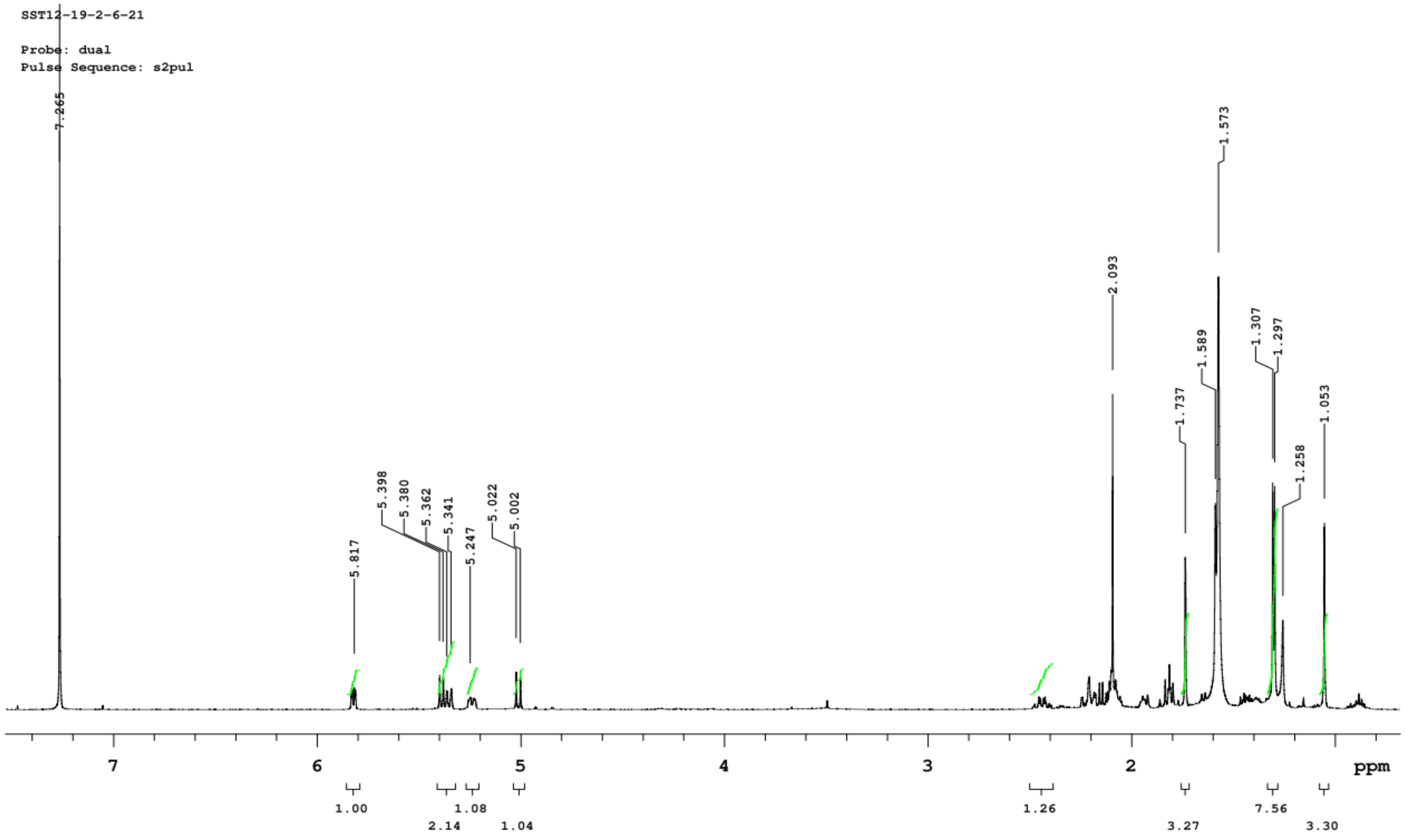

Figure S11. ^{13}C NMR spectrum (500 MHz) of numerosol B (**2**) in $CDCl_3$.

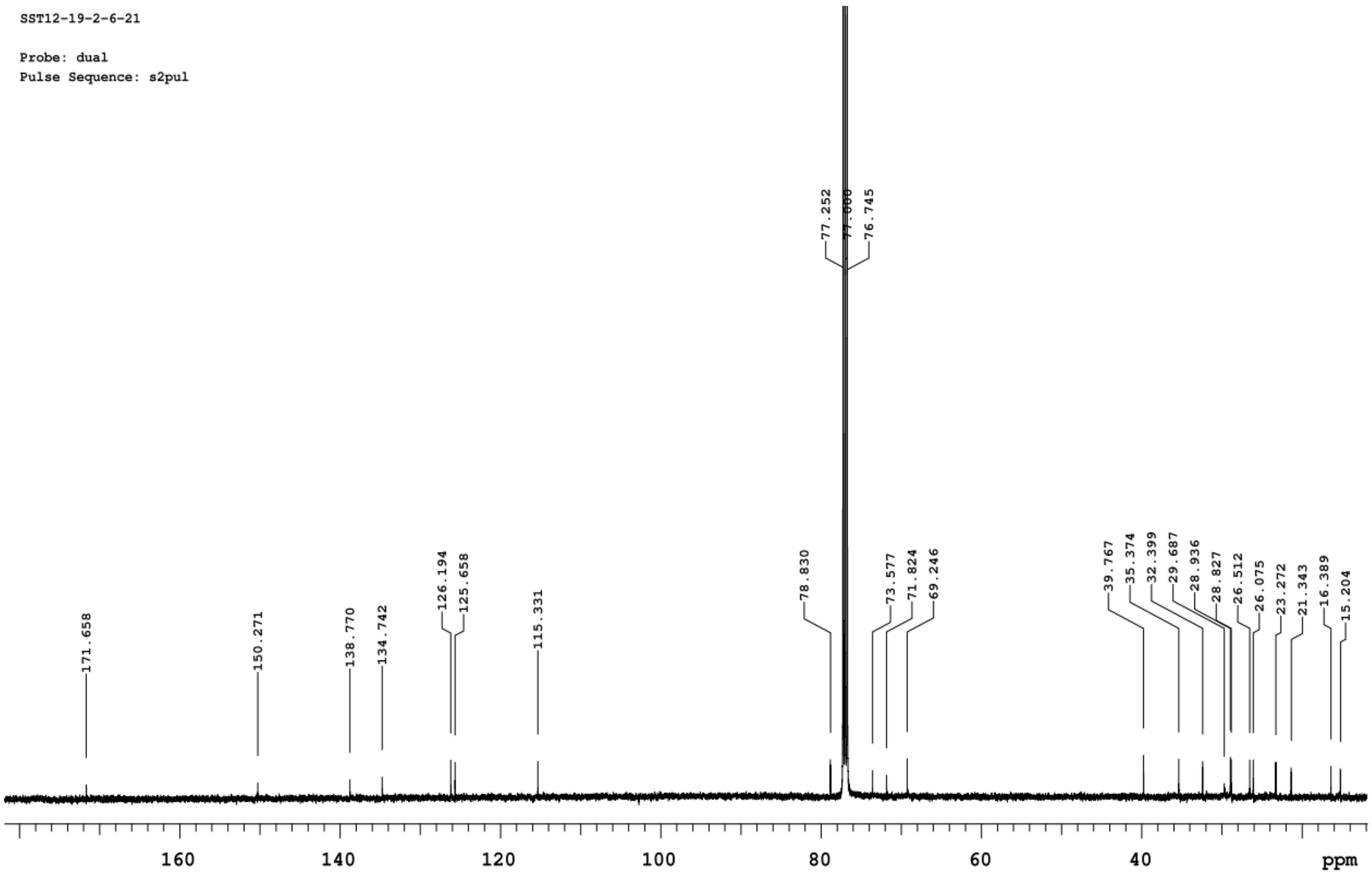

Figure S12. COSY spectrum (500 MHz) of numerosol B (**2**) in $CDCl_3$.

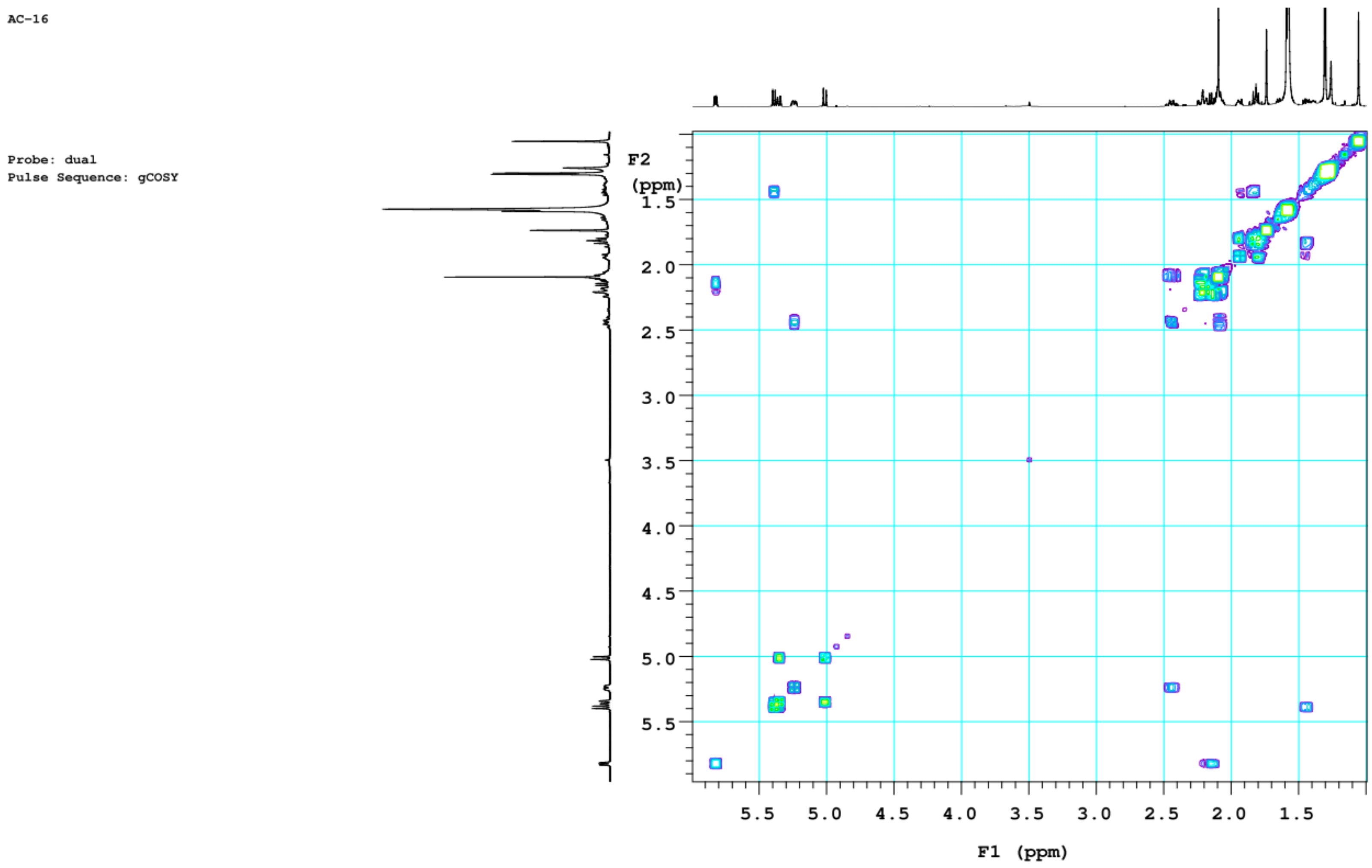

Figure S13. HSQC spectrum (500 MHz) of numerosol B (**2**) in $CDCl_3$.

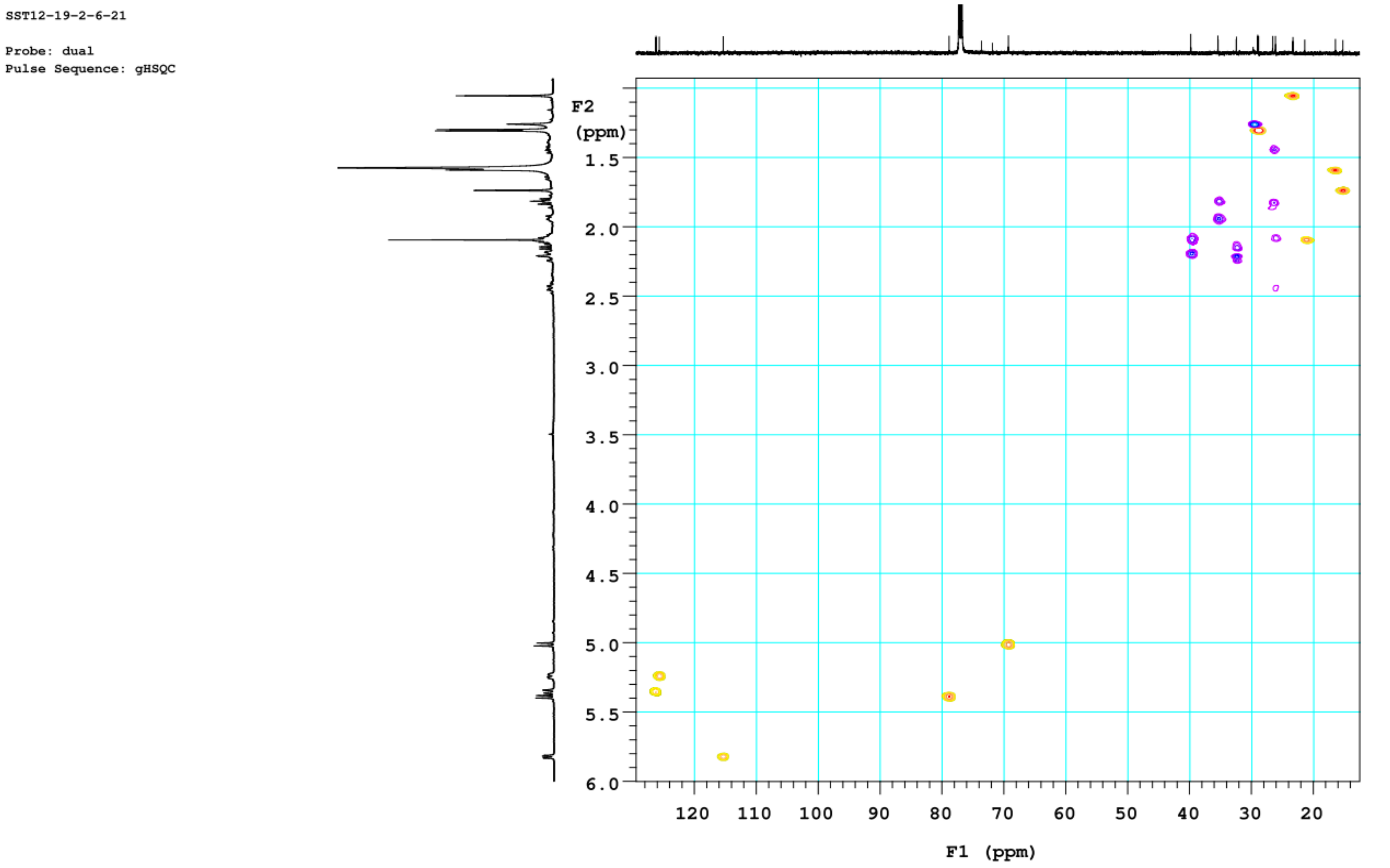

Figure S14. HMBC spectrum (500 MHz) of numerosol B (**2**) in $CDCl_3$.

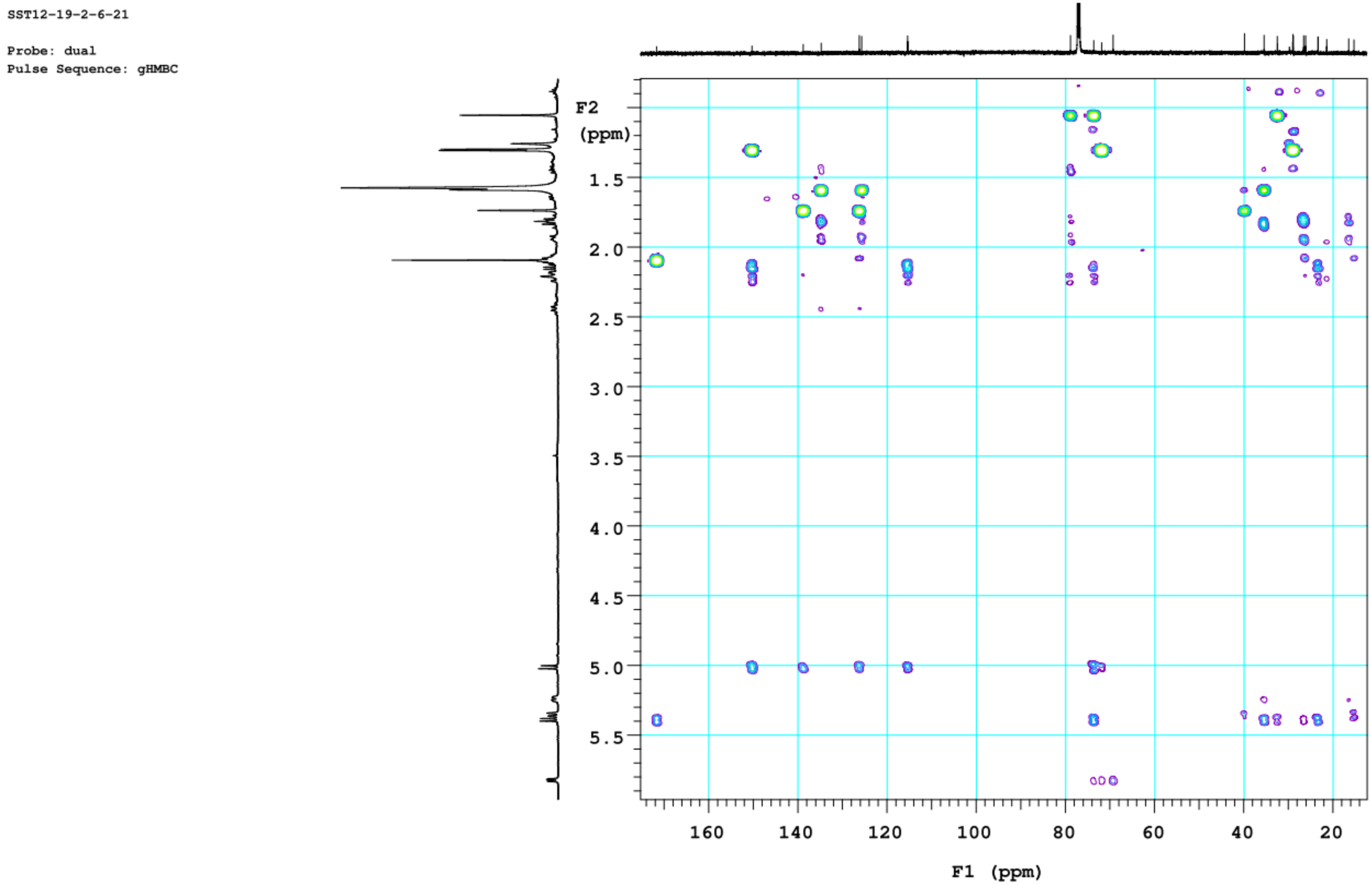

Figure S15. NOESY spectrum (500 MHz) of numerosol B (**2**) in $CDCl_3$.

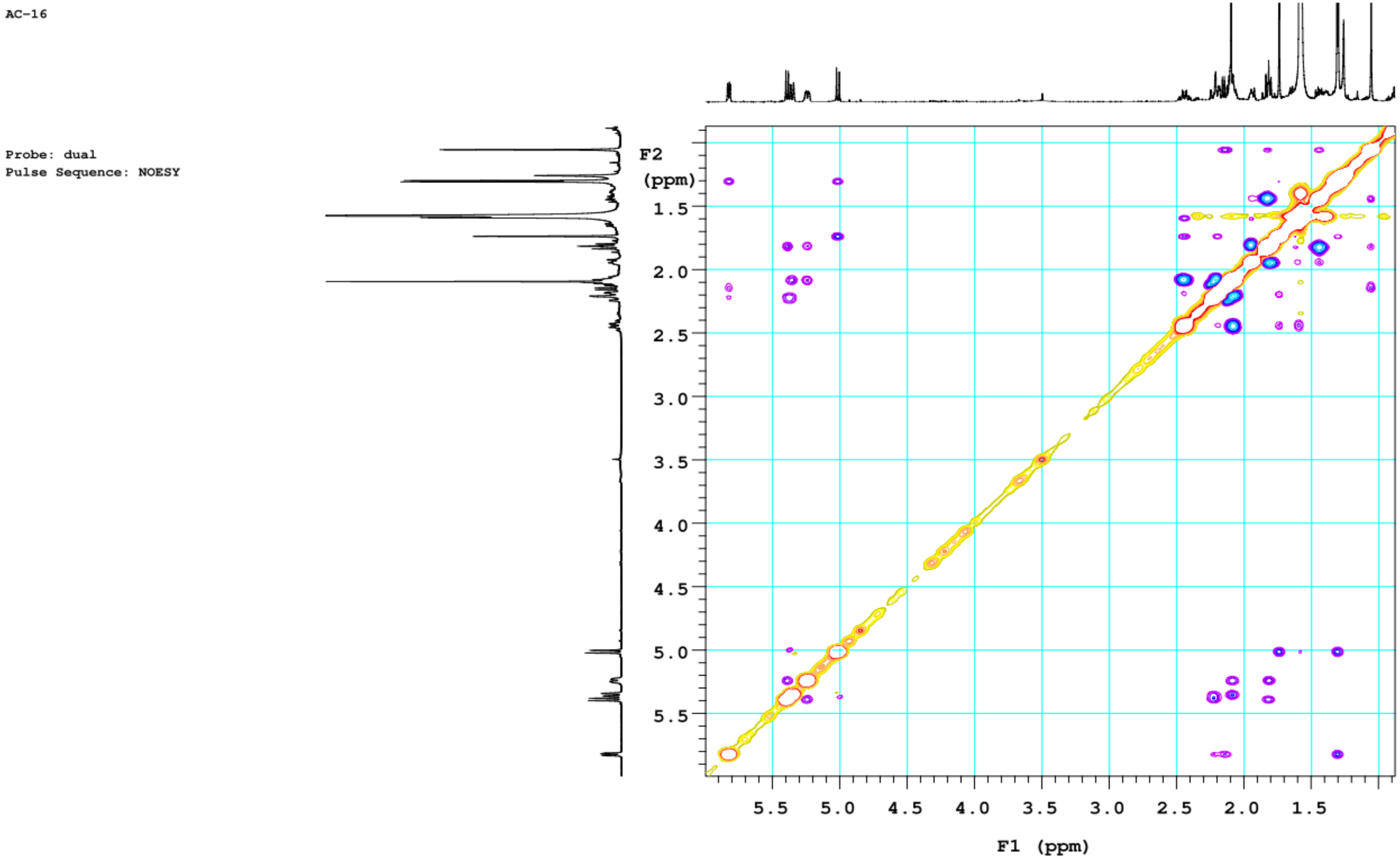

Figure S16. ^{1}H NMR spectrum (400 MHz) of numerosol C (**3**) in $CDCl_3$.

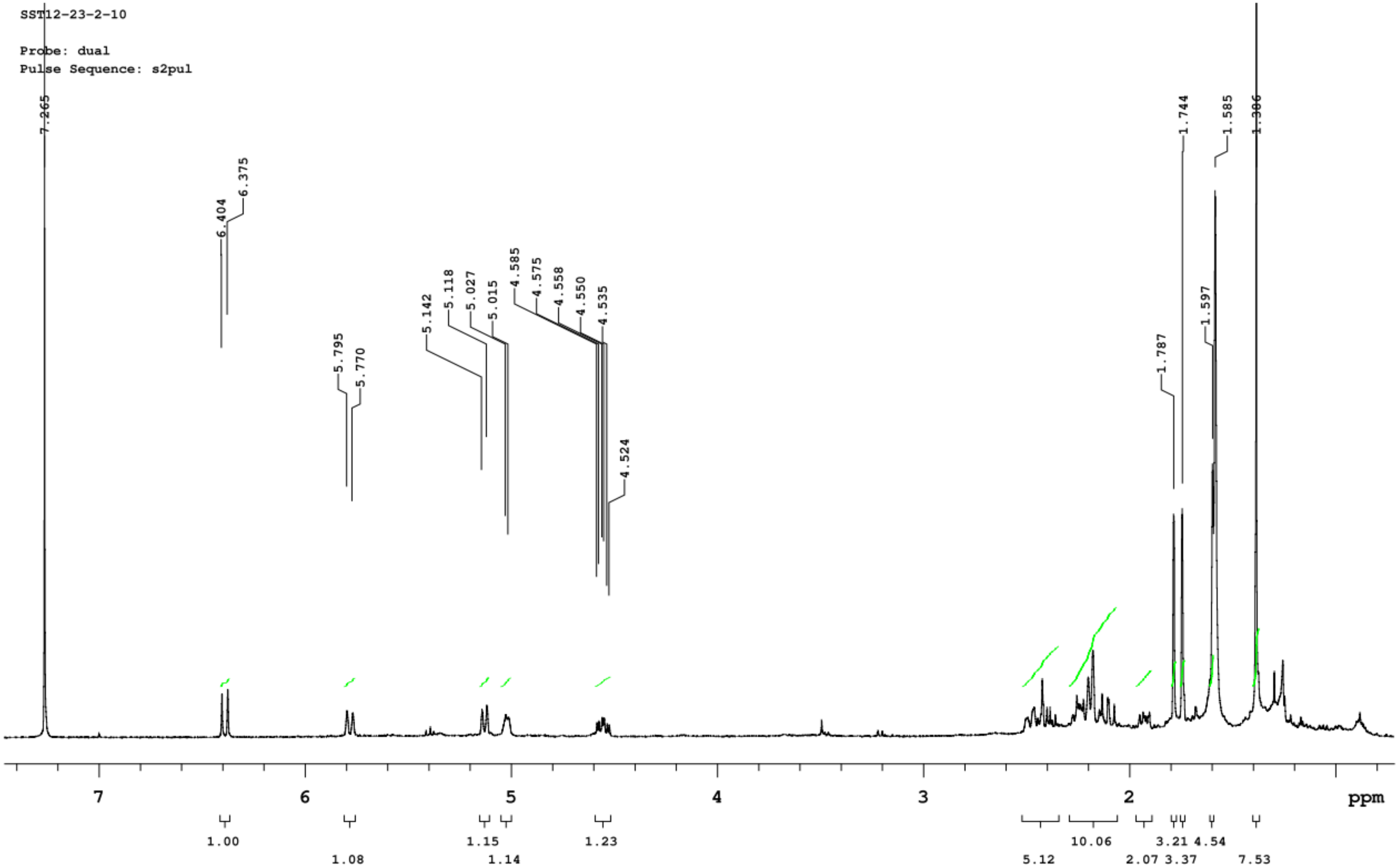

Figure S17. ^{13}C NMR spectrum (400 MHz) of numerosol C (**3**) in $CDCl_3$.

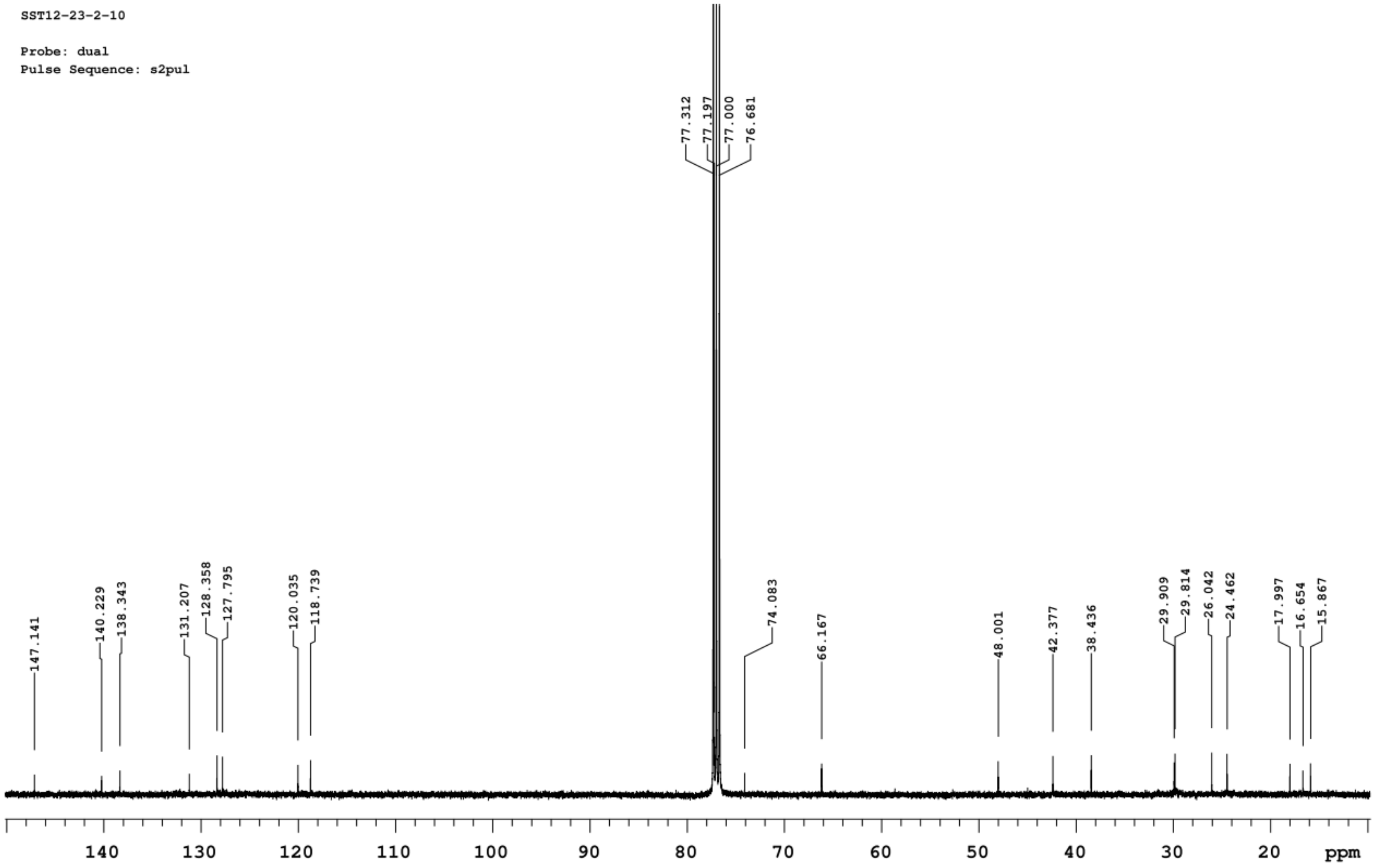

Figure S18. COSY spectrum (400 MHz) of numerosol C (**3**) in $CDCl_3$.

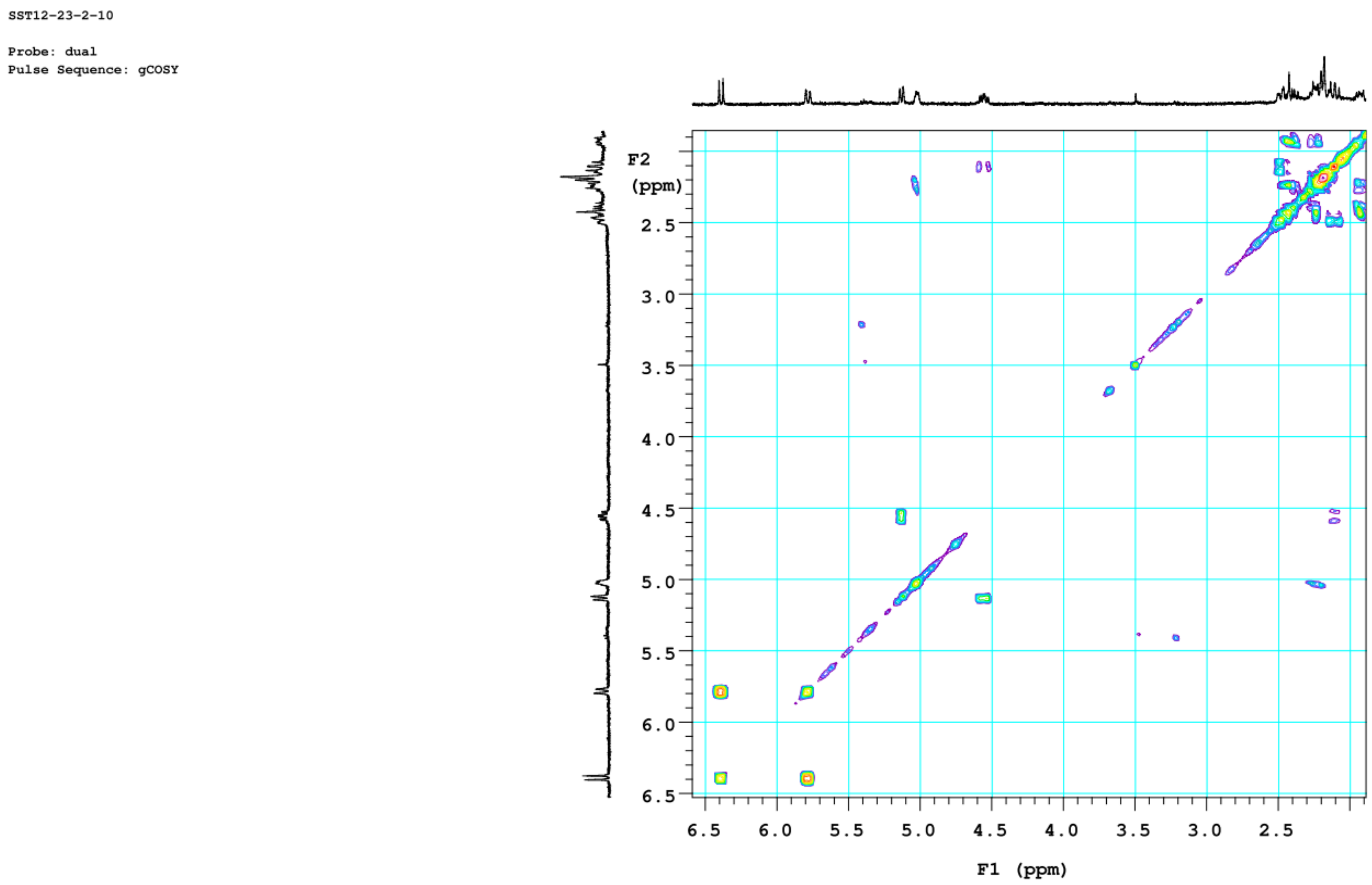

Figure S19. HSQC spectrum (400 MHz) of numerosol C (**3**) in $CDCl_3$.

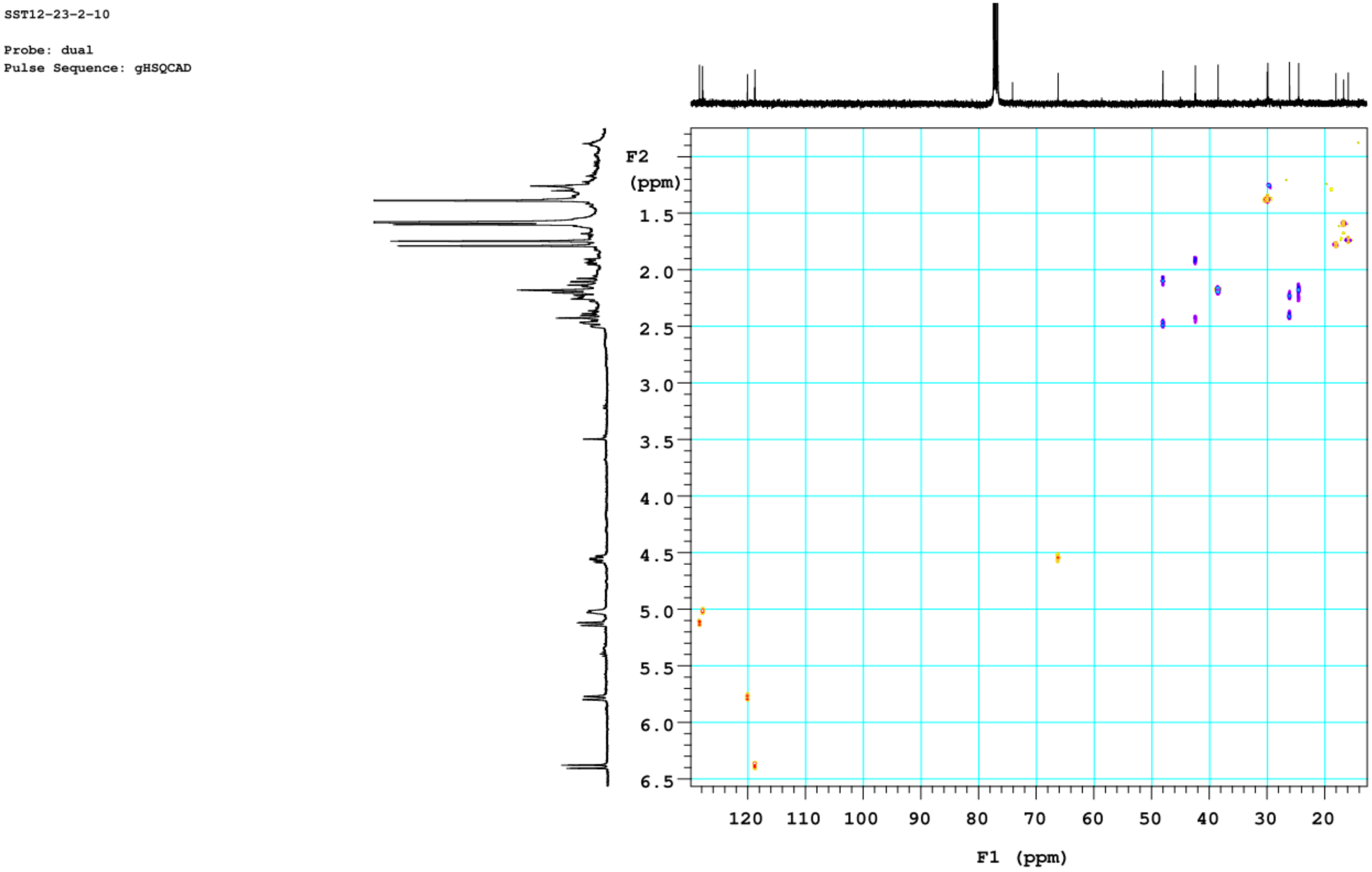

Figure S20. HMBC spectrum (400 MHz) of numerosol C (**3**) in $CDCl_3$.

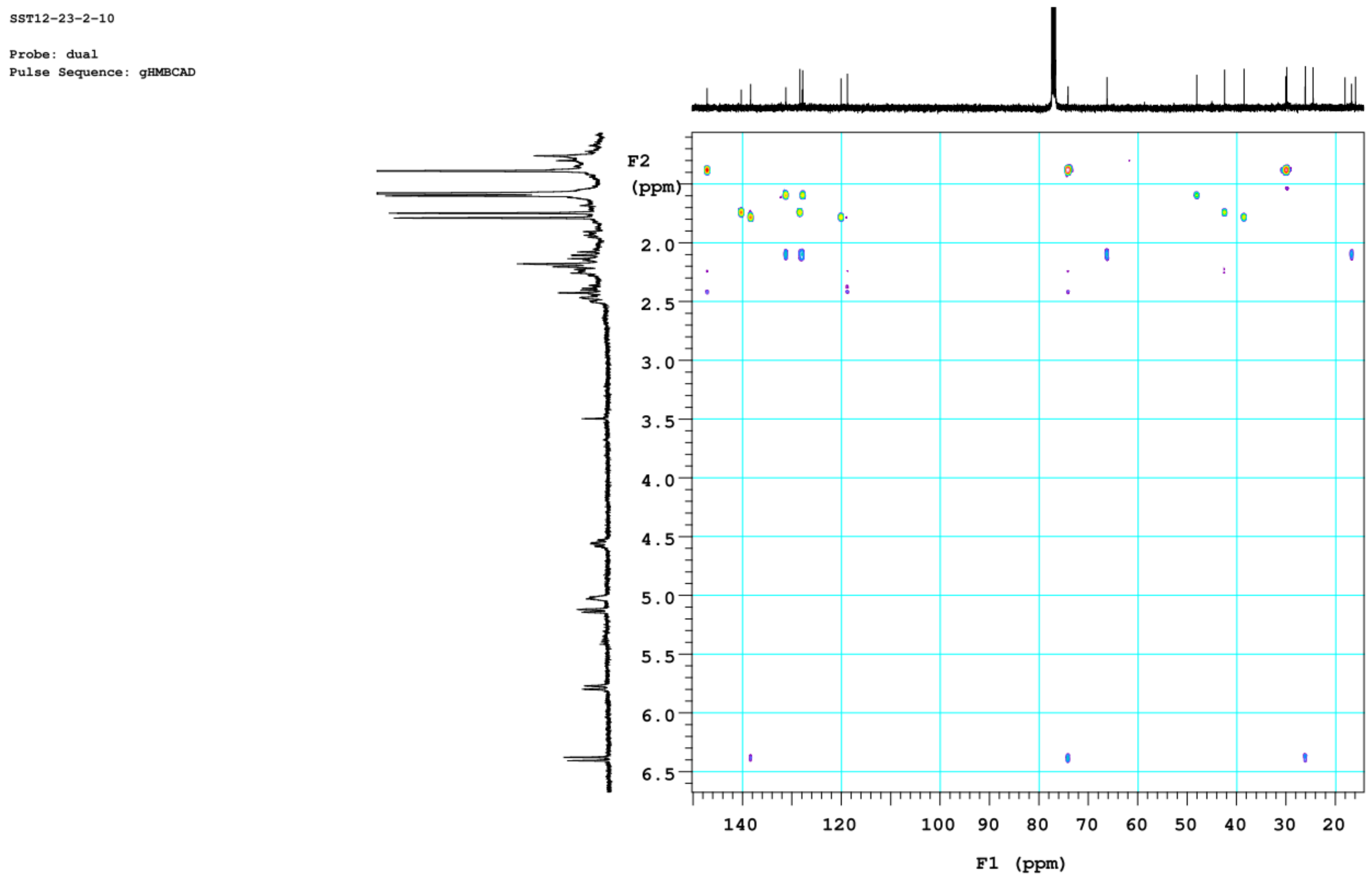

Figure S21. NOESY spectrum (400 MHz) of numerosol C (**3**) in $CDCl_3$.

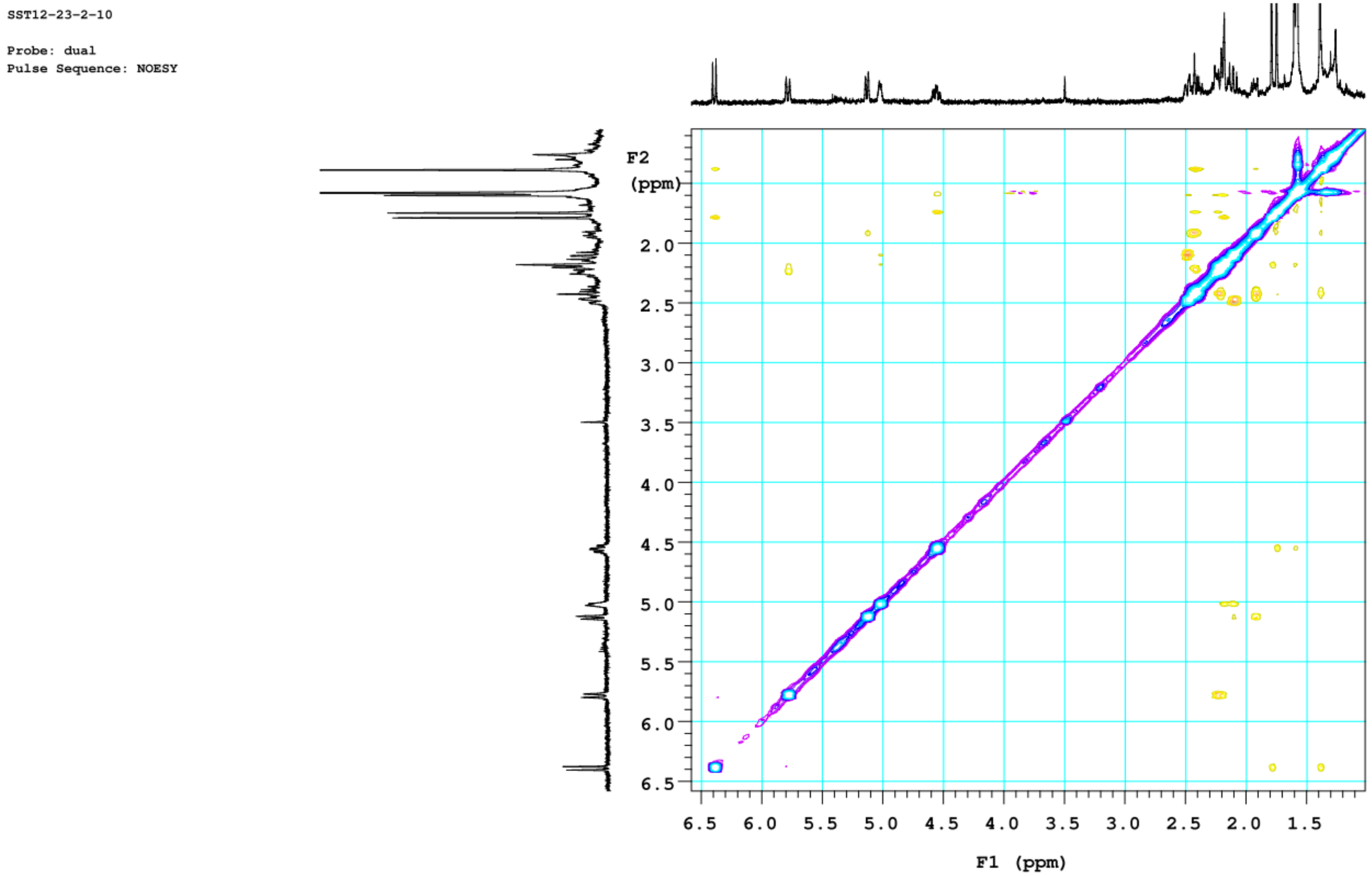

Figure S22. ^{1}H NMR spectrum (400 MHz) of numerosol D (**4**) in $CDCl_3$.

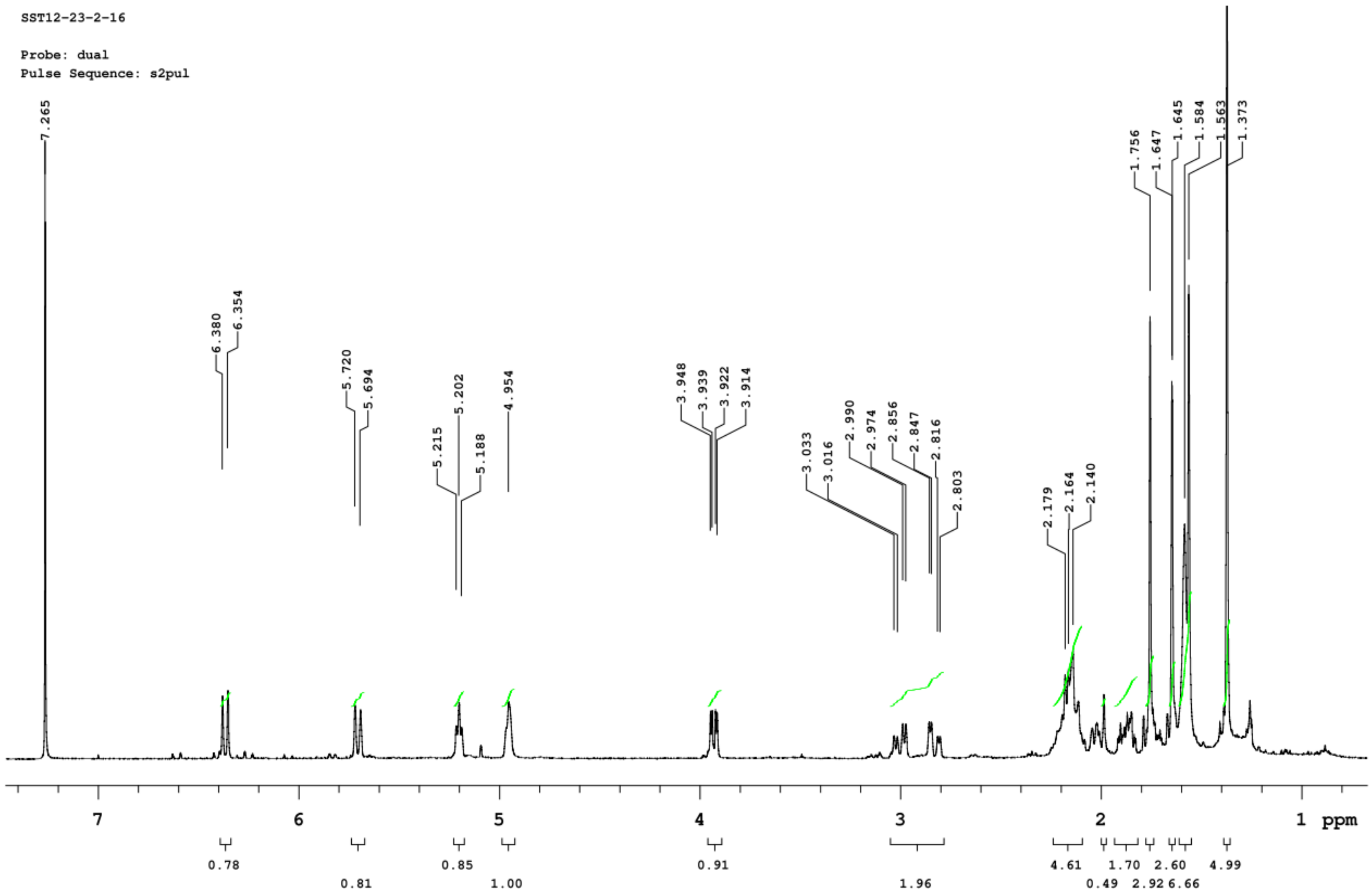

Figure S23. ^{13}C NMR spectrum (400 MHz) of numerosol D (**4**) in $CDCl_3$.

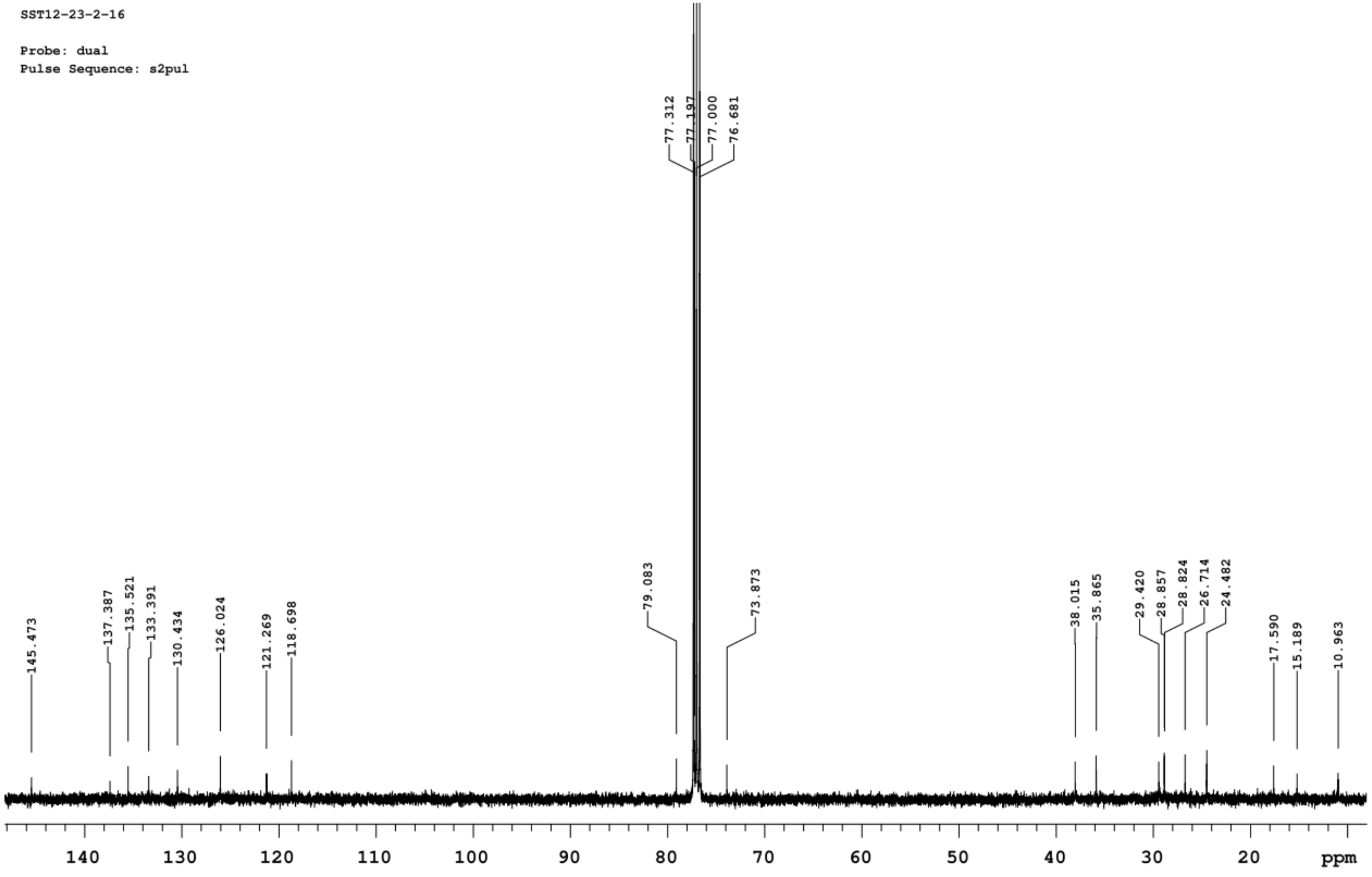

Figure S24. COSY spectrum (400 MHz) of numerosol D (**4**) in $CDCl_3$.

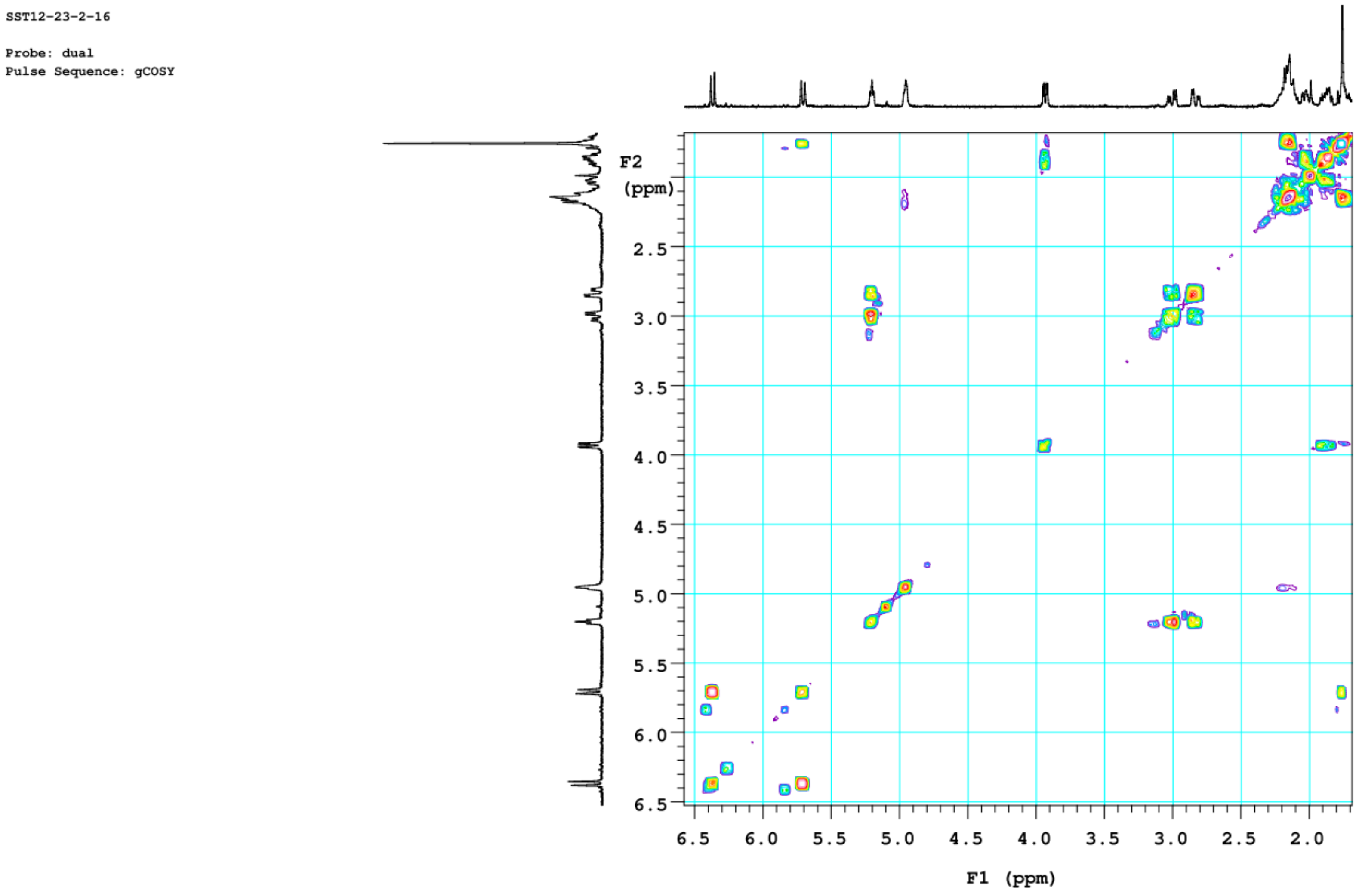

Figure S25. HSQC spectrum (400 MHz) of numerosol D (**4**) in $CDCl_3$.

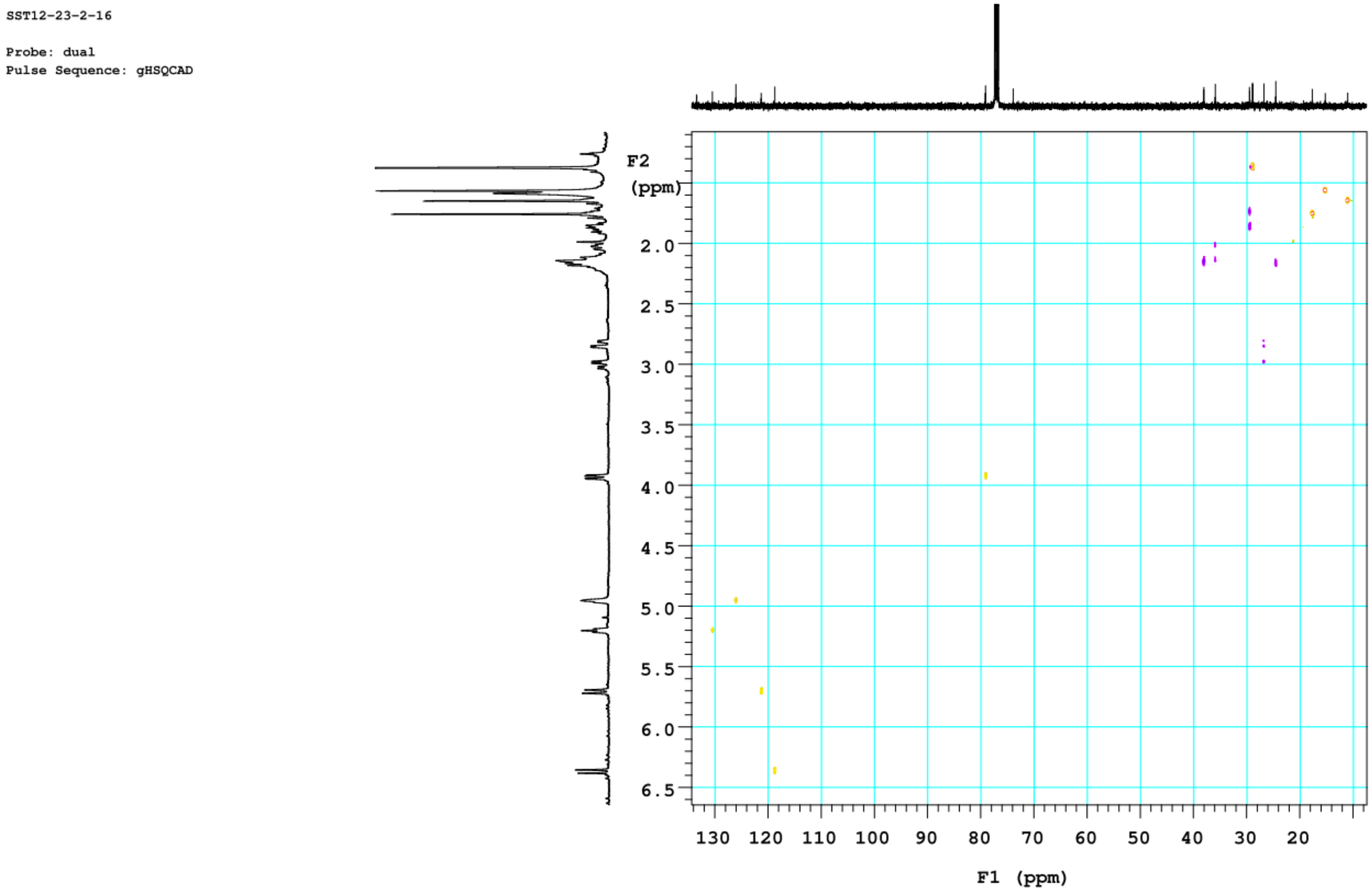

Figure S26. HMBC spectrum (400 MHz) of numerosol D (**4**) in $CDCl_3$.

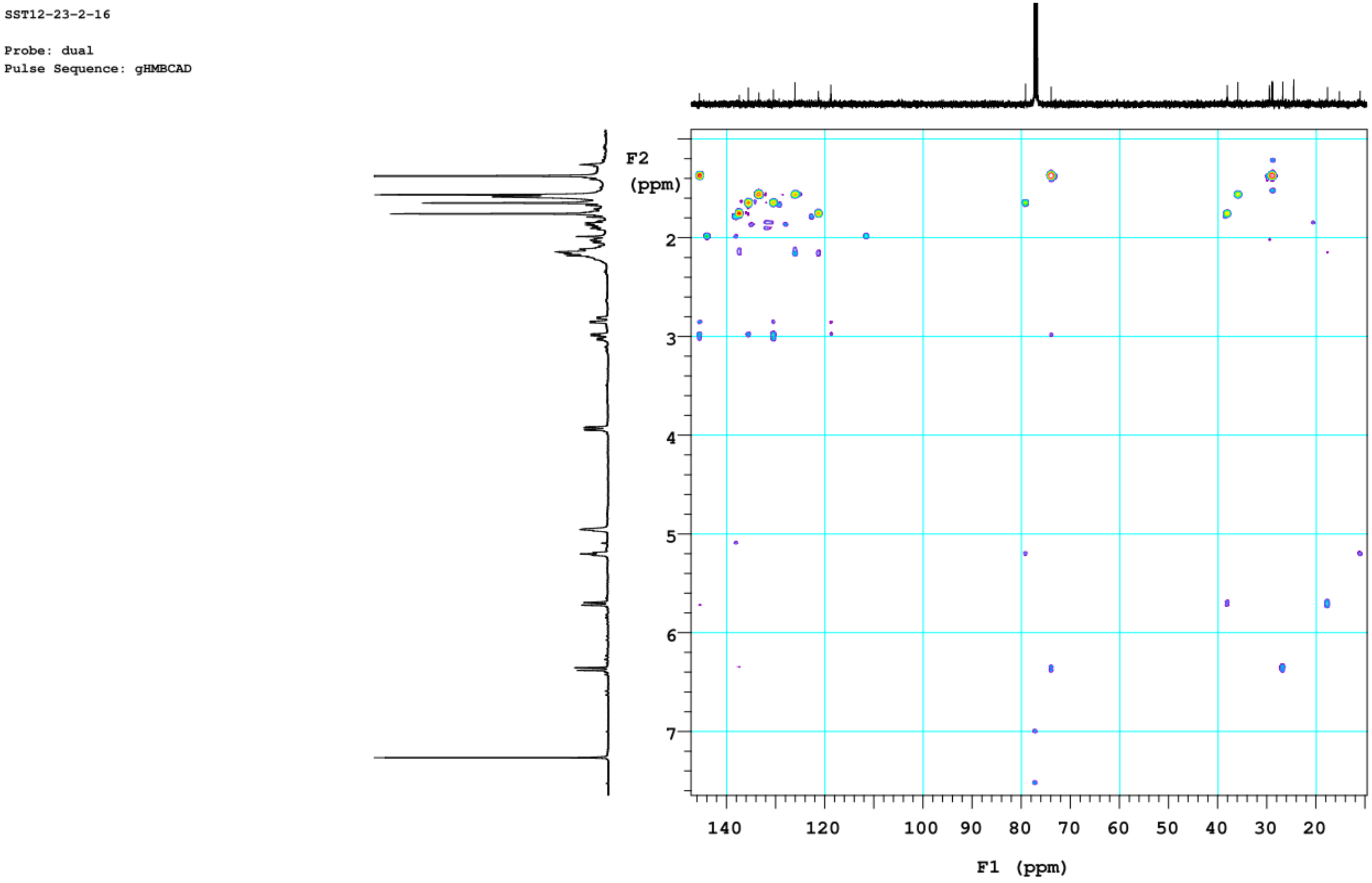

Figure S27. NOESY spectrum (400 MHz) of numerosol D (**4**) in $CDCl_3$.

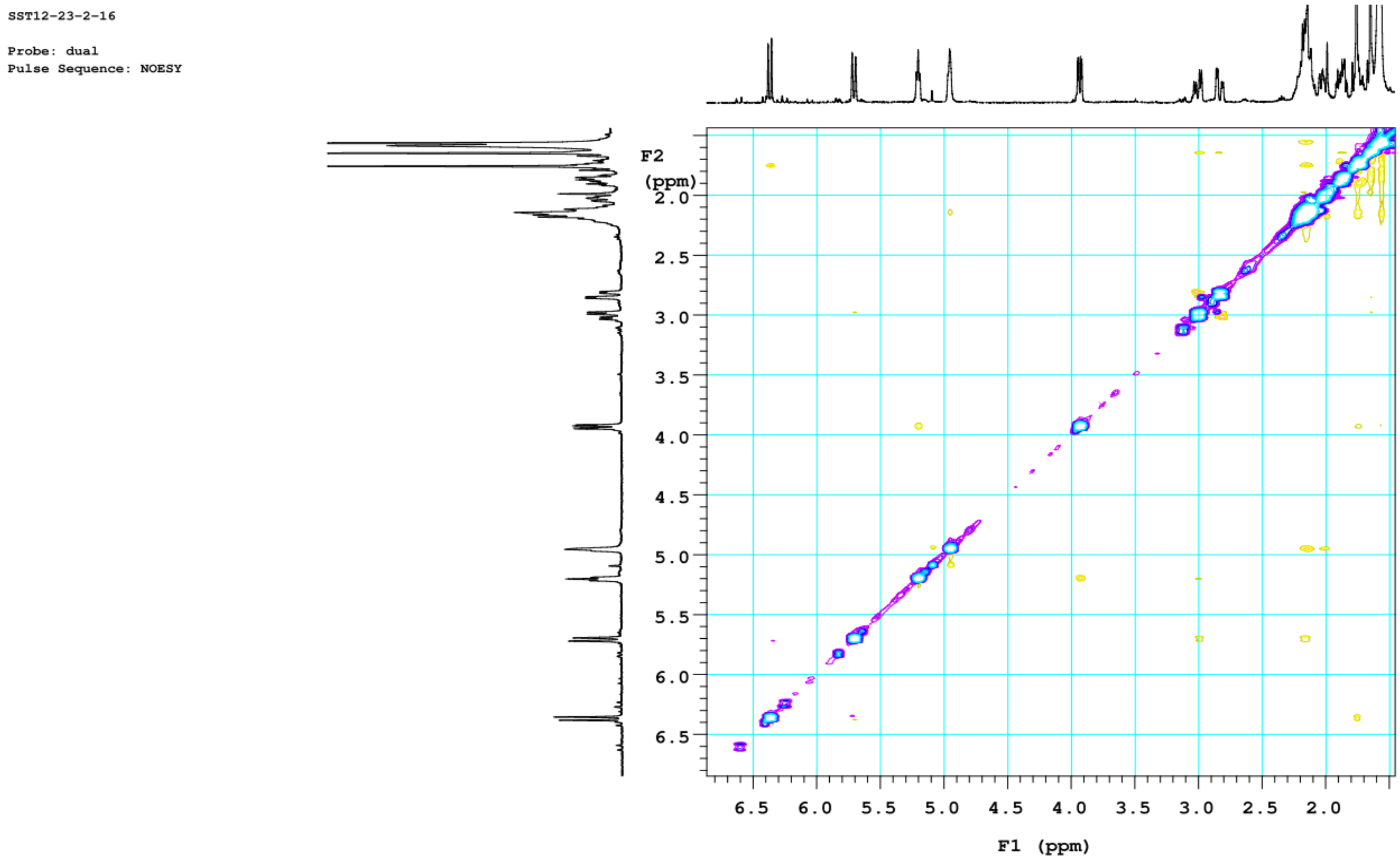

© 2014 by the authors; licensee MDPI, Basel, Switzerland. This article is an open access article distributed under the terms and conditions of the Creative Commons Attribution license (http://creativecommons.org/licenses/by/3.0/).
